# Investigating neuropathological correlates of hyperactive and psychotic symptoms in dementia: a systematic review

**DOI:** 10.3389/frdem.2025.1513644

**Published:** 2025-01-29

**Authors:** Giulia Negro, Michele Rossi, Camillo Imbimbo, Alberto Gatti, Andrea Magi, Ildebrando Marco Appollonio, Alfredo Costa, Tino Emanuele Poloni

**Affiliations:** ^1^Neurology Department, Fondazione IRCCS San Gerardo dei Tintori, San Gerardo Hospital, Monza, Italy; ^2^School of Medicine and Surgery and Milan Centre for Neuroscience (NeuroMI), University of Milano-Bicocca, Milan, Italy; ^3^Unit of Biostatistics, Golgi-Cenci Foundation, Abbiategrasso, Milan, Italy; ^4^Department of Brain and Behavioral Sciences, University of Pavia, Pavia, Italy; ^5^Unit of Behavioral Neurology and Center for Cognitive Disorders and Dementia (CDCD), IRCCS Mondino Foundation, Pavia, Italy; ^6^Department of Neurology and Neuropathology, Golgi-Cenci Foundation, Abbiategrasso, Milan, Italy; ^7^Department of Rehabilitation, ASP Golgi-Redaelli, Abbiategrasso, Milan, Italy

**Keywords:** neuropathology, BPSD, neuropsychiatric symptoms, psychosis, dementia, Alzheimer, Lewy bodies, FTLD

## Abstract

**Introduction:**

Behavioral and Psychological Symptoms of Dementia (BPSD) are common neuropsychiatric manifestations that complicate the clinical course of dementia and impact caregiving. Among these, the Hyperactivity–Impulsivity–Irritiability–Disinhibition–Aggression–Agitation (HIDA) and Psychosis (P) domains are particularly challenging to manage. Despite their prevalence, their underlying mechanisms and neuropathological correlates, remain poorly understood. This systematic review aims to elucidate the neuropathological basis of the HIDA and psychosis domains, exploring whether distinct proteinopathies and neural circuit dysfunctions are associated with these symptoms.

**Methods:**

The review follows PRISMA guidelines, with a systematic search conducted across MEDLINE, CENTRAL, and EMBASE databases. Inclusion criteria involved studies exploring the neuropathology of the HIDA and psychosis domains in individuals with dementia. Records were screened using PICO software, and data quality was assessed using the Newcastle-Ottawa Scale (NOS) and CARE guidelines. A narrative synthesis was conducted due to heterogeneity in the data.

**Results:**

From 846 records identified, 37 studies met inclusion criteria. Of the 18,823 cases analyzed, the most common diagnoses were Alzheimer's Disease (83.44%), Dementia with Lewy Bodies (5.37%), and Frontotemporal Dementia (13.40%). HIDA-P symptoms were distributed across all clinical diagnoses, with agitation (14.00%), delusions (11.60%), disinhibition (7.61%), and hallucinations (6.83%) being the most frequently reported behaviors. The primary neuropathological diagnosis was Alzheimer's Disease Neuropathologic Change (ADNC), present predominantly in intermediate to severe forms. The neuropathological analysis revealed the co-occurrence of multiple proteinopathies, particularly TAUopathy, TDP-43 pathology, and Lewy-related pathology (LRP), with the latter, in association with ADNC, reported in 15 studies.

**Discussion:**

HIDA-P symptoms were linked with overlapping involvement of different neural circuits, particularly the amygdala and the broader limbic system. Evidence suggests that TAUopathy and multiple proteinopathies in key brain regions, such as amygdala, are central to the development of these symptoms. In contrast, the contribution of beta-amyloid and vascular damage appears marginal in the genesis of HIDA and psychotic symptoms. No behavioral symptom is pathognomonic of a specific proteinopathy; rather, the topography and severity of lesions plays a more decisive role than their single molecular composition.

**Systematic review registration:**

INPLASY2024100082.

## 1 Introduction

According to the current definition, major Neuro-Cognitive Disorder (NCD), which corresponds to dementia, is a clinical syndrome characterized by a significant cognitive decline in one or more cognitive domains interfering with the independence of activities of daily living (American Psychiatric Association, 2013). This definition emphasizes that the disorder results from dysfunction within specific underlying neuronal circuits. In addition to cognitive impairment, neuropsychiatric symptoms, also referred to as Behavioral and Psychological Symptoms of Dementia (BPSD), are highly prevalent and significantly complicate the clinical course of dementia. Although already known for a long time under various nomenclatures (e.g., non-cognitive symptoms, neuropsychiatric symptoms), the term BPSD was defined and formalized in 1996 during the consensus conference held by the International Psychogeriatric Association (IPA) (Finkel, 2000). In 2013, the Diagnostic and Statistical Manual of Mental Disorders, Fifth Edition (DSM-5), highlighted these symptoms by including them as specifiers within the diagnostic criteria for neurodegenerative diseases. These specifiers include agitation, anxiety, mood symptoms (e.g., dysphoria, irritability, and euphoria), psychotic disturbances (e.g., hallucinations and delusions), and behavioral and psychological disturbances (e.g., apathy, aggression, disinhibition, disruptive behaviors or vocalizations, and disturbances in sleep or appetite/eating) (American Psychiatric Association, 2013). BPSD can be found in almost all types of dementia, each of which has distinct characteristics depending on the specific form. Further, some of these symptoms constitute core diagnostic criteria for different types of dementia such as Dementia with Lewy Bodies (DLB - i.e., recurrent visual hallucinations and rapid eye movement - REM - sleep behavior disorders) (Mckeith et al., 2017) and behavioral variant of Frontotemporal Dementia (bvFTD - i.e. apathy, aberrant motor behavior, disinhibition, and eating disorders) (Rascovsky et al., 2011). At a certain stage in the clinical course, over 80% of individuals with cognitive impairment will exhibit BPSD (Lyketsos et al., 2002), which contribute significantly to the cost of dementia care (Herrmann et al., 2006). The importance of BPSD is further underscored by the definition of Mild Behavioral Impairment (MBI), which identifies the late-life onset of behavioral and psychological symptoms before cognitive impairment or at least during mild NCD (or Mild Cognitive Impairment - MCI) as an early indicator of neurodegenerative disease (Ismail et al., 2016). Despite their heterogeneity, certain neuropsychiatric symptoms frequently co-occur suggesting a possible common pathogenesis for symptoms in each domain (Keszycki et al., 2019). Consequently, various studies have categorized these symptoms into distinct clusters. Among these, Van Der Linde et al. conducted a systematic analysis of 62 studies utilizing unbiased clustering approaches. This analysis identified the following BPSD domains: the affective domain, the psychosis domain (including hallucinations and delusions), the apathy domain, and the euphoria and hyperactivity-impulsivity-irritability-disinhibition-aggression-agitation (HIDA) domain (Keszycki et al., 2019; Van der Linde et al., 2014). Notably, the psychosis and HIDA domains, which have a prevalence rate of 17% to 40%, pose considerable challenges in the management of dementia patients (Zhao et al., 2016a,b). As a matter of fact, the risk of institutionalization is higher for individuals who exhibit the psychosis and HIDA domains (Keszycki et al., 2019; Gilley et al., 2004), which are also associated with an increase in the distress of caregivers (Kim et al., 2021; Fischer et al., 2012; Cerejeira et al., 2012). Further, a systematic review of 59 studies analyzing the longitudinal course of BPSD showed that psychosis, hyperactivity, agitation and physical aggression were associated with greater cognitive impairment (Van der Linde et al., 2016). Several neuroimaging studies were carried out to elucidate the pathophysiology of BPSD, highlighting the involvement of various brain regions and networks, particularly the default mode network and the limbic system, which consist of largely overlapping circuits (Cotta Ramusino et al., 2024; Zhao et al., 2024). Phosphorylated tau deposits, independent of amyloid load, and microglial activation have emerged as the most significant alterations observed in positron emission tomography (PET) scans (Tissot et al., 2021; Schaffer Aguzzoli et al., 2023). However, the underlying neuropathological correlates remain largely unexplored. Given that certain BPSD are integral to the core diagnostic criteria of various NCDs, their neuropathological basis may depend on the specific proteinopathy involved (i.e. visual hallucinations caused by Lewy Type Synucleinopathy - LTS - in patients with DLB) or on the dysfunction of particular underlying neural circuits (e.g., limbic system dysfunction leading to disinhibition in patients with Fronto-Temporal Lobar Degeneration - FTLD). Alongside pure neurodegenerative diseases, mixed pathologies are frequently identified, which involve the same neuronal circuits and complicate the accurate definition of the underlying basis of BPSD due to the numerous possible combinations of proteinopathies (Rahimi and Kovacs, 2014; Clinton et al., 2010). Therefore, what is the role of combined pathologies and their topographical distribution in this context? Further, due to the limited number of studies, some of which present contradictory findings, it is challenging to conduct a comprehensive analysis of the available data concerning the neuropathology of BPSD. This is particularly true for the psychosis and HIDA domains, which have unfavorable prognostic implications and impose a significant burden on caregivers, thereby representing the greatest therapeutic challenge (Cerejeira et al., 2012).

Given that over 55 million people worldwide are living with dementia, with projections indicating this number will triple by 2050 (World Health Organization, 2024), and considering the high prevalence of BPSD throughout the disease course (Lyketsos et al., 2002), a comprehensive understanding of the mechanisms underlying neuropsychiatric symptoms is crucial, also for developing more effective therapeutic strategies. The most straightforward and logical approach to elucidating the pathophysiological mechanisms of BPSD is through clinical-neuropathological correlations. This systematic review aims to summarize the current evidence regarding the neuropathological basis underlying the HIDA and psychosis (HIDA-P) domains of BPSD. Specifically, it seeks to elucidate whether distinct proteinopathies are associated with various individual symptoms within the HIDA-P domains. Furthermore, the review investigates whether these proteinopathies spread according to specific patterns throughout the brain, affecting distinct neural circuits depending on the symptoms.

## 2 Materials and methods

### 2.1 Literature search method

The results were reported following the Preferred Reporting Items for Systematic Reviews and Meta-Analyses (PRISMA) guidelines (Moher et al., 2009; Page et al., 2021). Studies were identified up to 7 March 2024 by a systematic search of the MEDLINE (accessed by PubMed), Cochrane Central Register of Controlled Trials (CENTRAL), and EMBASE databases. PICO frame [patients (P)/intervention (I)/comparison (C)/outcome (O)] was used for the search strategy: P = major/mild neurocognitive disorder associated with BPSD belonging to the HIDA cluster and/or psychosis domain/I = neuropathological assessment/C = not applicable/O = not applicable. The essential search terms included “behavioral and psychological symptoms,” “hyperactivity,” “impulsivity,” “irritability,” “disinhibition,” “aggression,” “agitation,” “dementia,” “delusion,” “hallucination,” and “neuropathology;” detailed search strategies and PRISMA checklist are provided in Appendix I. Searches were limited to human studies and English language articles without any date restriction. Conference papers, posters, abstracts, letters to the author, editorials, and reviews were excluded. The reference list of retrieved studies was reviewed to identify additional articles. The review protocol has been registered in the International Platform of Registered Systematic Reviews and Meta-analysis Protocol (INPLASY2024100082).

### 2.2 Eligibility criteria

The inclusion criteria involved studies whose aim is to examine neuropathologically the HIDA cluster and/or psychosis domain in human subjects with dementia. Studies attributing BPSD to an underlying psychiatric diagnosis were excluded from the analysis.

### 2.3 Study selection, data extraction, quality assessment, and ethic compliance

The PICO portal (automation tool software version 3.0.2023.1205) was used to screen the imported records. Three reviewers independently screened the title and abstract of the records identified by the literature search using the selected database and the search strategy. Disagreements were solved through a consensus meeting among the reviewers. Three authors performed the data extraction and the quality assessment of the included studies by using the Newcastle–Ottawa Scale (NOS) (Lo et al., 2014) and the CARE Guidelines (Gagnier et al., 2013) accessed on 17 July 2024. The following data were extracted from the included articles: main study author, year of publication, country where the research was conducted, number and demographic of subjects, clinical diagnosis, HIDA-P, and neuropathological assessment. This manuscript is based on previously performed studies and contains no new studies with human participants or animals.

### 2.4 Statistical analysis

A narrative synthesis of data extracted from the eligible records was carried out to summarize the information. The relevant measures were extracted, harmonized, and summarized into descriptive statistics, including the number (n), mean, and standard deviation (SD) for continuous variables, and frequencies and percentages for categorical variables. General descriptive statistics were presented as weighted means and weighted standard deviations (SD_W_). A meta-analysis could not be performed due to the considerable heterogeneity in the data reported across the included studies, which prevented the identification of uniform variables necessary for inferential statistical analysis.

## 3 Results

### 3.1 Results of the literature search

**Figure 1:**
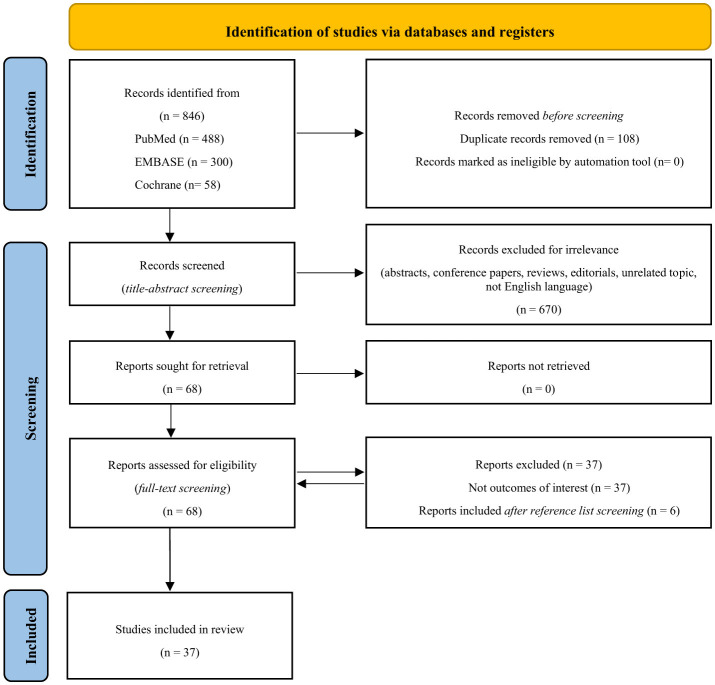
The study selection process using the PRISMA flow diagram.

The PRISMA flow diagram outlines the detailed plan of the structured literature search and selection process (Figure 1) (Page et al., 2021). The literature search yielded a total of 846 records. After the removal of duplicates and irrelevant articles, 68 full-text articles were assessed for eligibility. Six additional articles were identified through a review of reference lists from relevant studies. In total, 37 articles met the inclusion criteria. The reviewers involved in the study selection process reported a strong concordance of over 90% on including the records.

### 3.2 Quality assessment

The quality assessment results, evaluated using the Newcastle–Ottawa Scale (NOS) for case-control and cohort studies, an adapted version of the NOS for cross-sectional studies, and the CARE guidelines for case reports and case series, are provided in Appendix II (Lo et al., 2014; Gagnier et al., 2013). The assessment of the included research studies demonstrated a predominance of high-quality scores, with evaluations of 7/9, 8/9, and 9/9. Nearly all case-control and cohort studies satisfied the Newcastle-Ottawa Scale (NOS) criteria for selection, comparability, and exposure. In contrast, cross-sectional studies predominantly exhibited shortcomings in comparability and the statistical definition of sample size, identified as the main factors contributing to their lower quality. Consequently, 5 of these studies received moderate scores (4/8, 5/8, 6/8), while the remaining four were classified as high quality. High-quality scores were prevalent for case reports and case series, with ratings of 13/19, 14/19, and 18/19. However, two case reports received moderate quality scores of 11/19 and 12/19. The main factors affecting the quality of these studies included the definition of the title, description of therapeutic interventions, follow-up and outcomes, patient perspective, and the explicit collection of informed consent.

### 3.3 Characteristics of the studies included

Among the 37 articles included, 7 are case reports, one is a case series, and the remaining are research studies. Of the 29 studies, most were conducted in North America (*n* = 19), with a median publication year of 2017 (1991–2023). Most case reports and case series were described in Japan (*n* = 7), with a median publication year of 2018 (2005–2023) (Table 1). Regarding the study designs, 10 are cohort studies, 10 are categorized as case-control studies, and the remainder are cross-sectional studies.

**Table 1:** Summary of the general characteristics and demographic data of the articles included.

**Observational studies**							
**References**	**Country**	**Sample size, n**	**Male, n**	**Age at death, yrs, mean (SD)**	**Age at onset, yrs mean (SD)**	**Duration of illness, yrs mean (SD)**	**Education, yrs mean (SD)**
Ballard et al. (1999)	UK	80	40	79.85 (4.79)	74.90 (5.22)	4.95 (2.07)	*N/A*
Borges et al. (2019)	USA	887	452	79.81 (10.30)	69.96 (11.00)	9.35 (4.12)	15.04 (3.23)
Devanand et al. (2022)	USA	1,808	987	80.00 (11.00)	*N/A*	*N/A*	15.51 (3.11)
Echávarri et al. (2013)	Netherlands	80	35	77.75 (8.08)	70.75 (9.05)	7.00 (4.08)	*N/A*
Ehrenberg et al. (2018)	USA	455	236	70.50 (12.10)	*N/A*	*N/A*	4.70 (4.00)
Esteban de Antonio et al. (2020)	Spain	59	6	85.40 (6.60)	76.00 (7.50)	9.40 (9.99)	*N/A*
Farber et al. (2000)	USA	109	45	82.26 (8.20)	74,17 (9,01)	8.09 (3.73)	*N/A*
Ferman et al. (2013)	USA	125	62	80.12 (12.21)	69.34 (8.90)	10.78 (5.01)	*N/A*
Förstl et al. (1994)	UK	56	13	83.10 (6.20)	75.40 (7.40)	7.70 (4.60)	*N/A*
Garcia-Segura et al. (2019)	Canada	2,959	1,375	*N/A*	*N/A*	*N/A*	*N/A*
Gauthreaux et al. (2022)	USA	1,100	217	83.20 (8.80)	*N/A*	*N/A*	15.90 (3.10)
Gibson et al. (2023)	UK	1,038	531	72.20 (12.70)	*N/A*	*N/A*	4.80 (4.03)
Harding et al. (2002)	Australia	47	*N/A*	74.04 (9.16)	63.44 (10.87)	10.60 (5.85)	*N/A*
Hirsch-Reinshagen et al. (2023)	Canada	58	28	63,92 (10.99)	57.63 (10.24)	6.29 (3.98)	*N/A*
Lai et al. (2010)	Singapore	27	14	81.00 (7.00)	71,50 (7,05)	9.50 (0.80)	*N/A*
Léger and Banks (2014)	USA	727	428	77.34 (10.54)	*N/A*	*N/A*	15,65 (8,22)
Liu et al. (2020)	UK	131	75	82.89 (9.06)	*N/A*	*N/A*	12.41 (3.36)
Murray et al. (2014)	USA	26	12	81.40 (8.10)	71.20 (9.40)	10.20 (12.41)	*N/A*
Nelson et al. (2023)	USA	368	158	84.40 (8.80)	*N/A*	*N/A*	*N/A*
Pillai et al. (2022)	USA	2,422	1,446	*N/A*	*N/A*	5.55 (2.79)	15.55 (3.07)
Scarioni et al. (2020)	Netherlands	150	162	67.69 (10.19)	59.31 (10.84)	8.38 (14.88)	*N/A*
Sennik et al. (2016)	Canada	1,716	944	79.42 (10.33)	*N/A*	*N/A*	15.18 (3.17)
Shaw et al. (2024)	USA	4,033	2,280	80.20 (10.90)	*N/A*	*N/A*	15.60 (3.00)
Sweet et al. (2000)	USA	24	9	77.00 (9.20)	66.00 (9.30)	11.00 (13.08)	*N/A*
Tekin et al. (2001)	USA	31	9	*N/A*	*N/A*	*N/A*	13.60 (2.00)
Tsuang et al. (2009)	USA	148	67	83.98 (6.50)	76.41 (7.06)	7.57 (9.60)	*N/A*
Victoroff et al. (1996)	USA	99	53	77.11 (8.81)	68,50 (8,82)	8.61 (0.50)	*N/A*
Vik-Mo et al. (2019)	Norway	47	26	75,88 (8,61)	73.57 (8.38)	2.31 (1.99)	*N/A*
Zubenko et al. (1991)	USA	13	4	68.80 (7.60)	64.40 (7.70)	5.50 (2.50)	*N/A*
**Case reports and case series**							
**References**	**Country**	**Sex**	**Age at death, yrs**	**Age at onset, yrs**	**Age at HIDA-P onset, yrs**	**Duration of illness, yrs**	**Education, yrs**
Asaoka et al. (2010)	Japan	M	79	72	72	7	*N/A*
Beck et al. (2022)	Japan	M	69	*N/A*	59	*N/A*	16
Fujishiro et al. (2015)	Japan	F	83	69	69	14	13
Hirano et al. (2020)	Japan	M	79	67	*N/A*	12	*N/A*
Iwasaki et al. (2018)	Japan	M	96	*N/A*	*N/A*	*N/A*	*N/A*
Kawakami et al. (2023)	Japan	M M	75 83	*N/A* 79	70 73	*N/A* 4	*N/A N/A*
Shibuya-Tayoshi et al. (2005)	Japan	M	58	58	57	0	13
Tartaglia et al. (2008)	UK	F	76	65	*N/A*	11	*N/A*

n, number of subjects; SD, standard deviation; yrs, years; *N/A*, data not available; M, male; F, female.

### 3.4 Demographic characteristics

The general characteristics and demographic data of the included articles are presented in Table 1. The total number of cases from the 29 studies amounts to 18,828, of which qualitative analysis was carried out for 18,823 subjects with available data. Of these cases, 9,714 (49.58%) are male, resulting in a male-to-female ratio of 1.07. The average years of education for the 18,823 patients with available data is 14.36 (±3.63 SD_W_). The mean age at dementia onset is 70.00 years (±9.77 SD_W_), the average disease duration is 6.88 years (±4.84 SD_W_), and the mean age at death is 76.55 years (±10.57 SD_W_). Additionally, the narrative analysis includes 9 cases from case reports and case series. Of these, 7 (77.78%) are male, yielding a male-to-female ratio of 0.78. The average years of education for the 7 subjects with available information is 14. The mean age at dementia onset is 68.33 years, with an average disease duration of 8 years and a mean age at death of 77.56 years. For the HIDA-P symptom cluster, the mean age at onset is 66.67 years, and the interval between the onset of these symptoms and death is 7.83 years.

### 3.5 Clinical and neuropsychological characteristics

Given that many studies focused on neuropathology, clinical diagnoses were reported in only 17 studies. Among the 5,642 subjects with available clinical diagnosis, 4,708 were diagnosed with Alzheimer's Disease (AD−83.45%), 303 with DLB (5.37%), and 756 with Fronto-Temporal Dementia (FTD−13.40%). Notably, as indicated by the percentages, some cases demonstrated overlapping clinical diagnoses. Specifically, in the study by Sennik et al. (2016), of the 1,716 cases with Alzheimer's Disease, 192 also had a clinical diagnosis of Lewy Body Dementia syndrome, and 230 were additionally diagnosed with Fronto-Temporal Dementia. Similarly, in the study by Victoroff et al. (1996), among the 94 Alzheimer's Disease patients, 5 were also associated with Vascular Dementia (VaD). In the study by Echávarri et al. (2013), among 40 patients diagnosed with VaD, 6 were classified as having mixed dementia, without specifying the individual contributing diagnoses. The MiniMental State Examination (MMSE) scores were reported in 9 out of 29 studies, with an average score of 13.76 (±8.10 SD_W_). The mean Clinical Dementia Rating (CDR) global score, available from 5 studies, is 1.76 (±0.97 SD_W_). Most of the studies included in the analysis (*n* = 16) utilized the 12-item Neuropsychiatric Inventory Questionnaire (NPI-Q) for neuropsychological assessment. All data on the clinical characteristics of the included studies are presented in Table 2. Concerning the HIDA domain, data derived from the 29 studies show the following: hyperactivity was mentioned in 2 articles, although no frequency data was provided; irritability was reported in 12 articles, encompassing 1,317 cases (7.03%); disinhibition was noted in 15 articles, covering 1,475 cases (7.87%); aggression was reported in 5 articles, involving 903 cases (4.80%); aberrant motor behavior was noted in 12 articles, covering 1,010 cases (5.39%); and agitation was reported in 14 articles, affecting 2,624 cases (14.00%). Impulsivity, however, was not reported in any of the studies. Regarding the psychotic symptoms, delusions were documented in 22 studies, involving 2,168 cases (11.60%), while hallucinations were reported in 24 studies, affecting 1,281 subjects (6.83%). Notably, the study by Murray et al. (2014) used the term “psychoses” to encompass the presence of hallucinations and/or delusions in 26 cases, without specifying their individual distribution. The summary of behavioral characteristics data from the included studies is presented in Table 3. On the other hand, among case reports and case series, 2 subjects were diagnosed with AD, 2 with senile psychosis and depression, 1 with unspecified major neurocognitive impairment, and 4 with FTD, of which 2 with the behavioral variant of FTD (Table 2). Among the 9 cases with available MMSE scores, Fujishiro et al. (2015) reported a score of 18/30 at the first visit, while Shibuya-Tayoshi et al. (2005) noted a score of 27/30. Asaoka et al. (2010) documented a score of 22/30, recorded 5 years before the patient's death. The methods used to assess behavioral disorders were detailed in 4 articles, which included the NPI-Q (*n* = 1), patient anamnesis (*n* = 2), and a generic neuropsychological interview (*n* = 1). The frequency of symptoms within the HIDA-P domains are as follows: hyperactivity was observed in 3 cases (33.33%), as were impulsivity, aggression and aberrant motor behavior. Irritability was present in 5 out of 9 cases (55.55%), and the same frequency was noted for disinhibition, agitation, delusions, and hallucinations (Table 3). Considering the overall data, HIDA-P disorders were distributed across all clinical diagnoses, with no distinct association to any specific condition. As expected, given their inclusion in diagnostic criteria, REM sleep behavior disorder and hallucinations were more frequently associated with LBD, while disinhibition was more commonly observed in FTD.

**Table 2:** Summary of the clinical characteristics of the articles included.

**Observational studies**						
**References**	**Sample size, n**	**AD, n**	**DLB, n**	**FTD, n**	**MMSE, mean (SD)**	**CDR, mean (SD)**
Ferrer et al. (2008)	80	*N/A*	*N/A*	*N/A*	11.20 (13.67)	*N/A*
Borges et al. (2019)	887	756	0	131	*N/A*	*N/A*
Devanand et al. (2022)	*N/A*	*N/A*	*N/A*	*N/A*	*N/A*	*N/A*
Echávarri et al. (2013)	80	35	4	2	*N/A*	2.85 (0.30)
Ehrenberg et al. (2018)	455	455	0	0	*N/A*	*N/A*
Esteban de Antonio et al. (2020)	59	44	9	0	*N/A*	*N/A*
Farber et al. (2000)	109	109	0	0	*N/A*	2.32 (0.97)
Ballard et al. (1999)	125	*N/A*	*N/A*	*N/A*	*N/A*	*N/A*
Förstl et al. (1994)	56	56	0	0	*N/A*	*N/A*
Garcia-Segura et al. (2019)	2,959	*N/A*	*N/A*	*N/A*	*N/A*	*N/A*
Gauthreaux et al. (2022)	1,100	766	72	49	*N/A*	*N/A*
Gibson et al. (2023)	*N/A*	*N/A*	*N/A*	*N/A*	*N/A*	*N/A*
Harding et al. (2002)	47	0	25	0	*N/A*	*N/A*
Hirsch-Reinshagen et al. (2023)	58	3	1	48	*N/A*	*N/A*
Lai et al. (2010)	*N/A*	*N/A*	*N/A*	*N/A*	4.50 (1.40)	*N/A*
Léger and Banks (2014)	727	578	0	149	*N/A*	*N/A*
Liu et al. (2020)	*N/A*	*N/A*	*N/A*	*N/A*	13.90 (8.66)	2.47 (0.86)
Murray et al. (2014)	26	26	0	0	9.90 (6.40)	*N/A*
Nelson et al. (2023)	*N/A*	*N/A*	*N/A*	*N/A*	*N/A*	*N/A*
Pillai et al. (2022)	*N/A*	*N/A*	*N/A*	*N/A*	*N/A*	*N/A*
Scarioni et al. (2020)	150	2	0	146	*N/A*	*N/A*
Sennik et al. (2016)	1,716	1,716	192	230	14.26 (8.01)	*N/A*
Shaw et al. (2024)	*N/A*	*N/A*	*N/A*	*N/A*	*N/A*	1.68 (0.98)
Sweet et al. (2000)	24	24	0	0	*N/A*	*N/A*
Tekin et al. (2001)	31	31	0	0	8.10 (5.60)	*N/A*
Tsuang et al. (2009)	*N/A*	*N/A*	*N/A*	*N/A*	13.10 (7.58)	*N/A*
Victoroff et al. (1996)	104	94	0	1	8.18 (7.47)	2.38 (0.97)
Vik-Mo et al. (2019)	*N/A*	*N/A*	*N/A*	*N/A*	24.55 (2.61)	*N/A*
Zubenko et al. (1991)	13	13	0	0	*N/A*	*N/A*
**Case reports and case series**	
**References**	**Clinical diagnosis**
Asaoka et al. (2010)	*N/A*
Beck et al. (2022)	bvFTD
Fujishiro et al. (2015)	AD
Hirano et al. (2020)	bvFTD
Iwasaki et al. (2018)	AD
Kawakami et al. (2023)	Senile psychosis/depression
Shibuya-Tayoshi et al. (2005)	FTD

N, number of subjects; SD, standard deviation; *N/A*, data not available; AD, Alzheimer's Disease; DLB, Dementia with Lewy Bodies; FTD, Fronto-Temporal Dementia; bvFTD, behavioral variant FTD; MMSE, MiniMental State Examination; CDR, Clinical Dementia Rating global score.

**Table 3:** Summary of the behavioral characteristics the articles included.

**References**	**Behavioral assesment tool**	**Hyperactivity (n)**	**Impulsivity (n)**	**Irritability (n)**	**Disinhibition (n)**	**Aggressivity (n)**	**Agitation (n)**	**Delusions (n)**	**Hallucinations (n)**	**Aberrant motor behavior (n)**
Ferrer et al. (2008)	MOUSEPAD								✓	✓ (36)
Borges et al. (2019)	NPI-Q				✓ (108)					
Devanand et al. (2022)	NPI-Q	✓		✓	✓		✓	✓ (692)	✓ (517)	✓
Echávarri et al. (2013)	NPI-Q			✓ (7)	✓ (11)		✓ (45)	✓ (9)	✓ (17)	✓ (13)
Esteban de Antonio et al. (2020)	NPI-Q			✓ (67)	✓ (19)			✓ (29)	✓ (34)	✓ (21)
Farber et al. (2000)	NPI-Q			✓	✓	✓		✓	✓	✓
Ballard et al. (1999)	Clinical interview			✓ (55)	✓ (13)		✓ (59)	✓ (65)	✓ (35)	✓ (40)
Förstl et al. (1994)	*N/A*								✓ (64)	
Garcia-Segura et al. (2019)	Neuropsychological evaluation							✓	✓	
Esteban de Antonio et al. (2020)	NPI-Q									✓ (736)
Gauthreaux et al. (2022)	Neuropsychological evaluation			✓ (352)	✓ (201)		✓ (286)	✓ (180)	✓ (157)	
Gibson et al. (2023)	NPI-Q			✓ (155)	✓ (48)		✓ (164)	✓ (75)	✓ (105)	✓ (67)
Harding et al. (2002)	NPI-Q								✓ (31)	
Hirsch-Reinshagen et al. (2023)	Medical records						✓ (3)	✓ (15)	✓ (5)	
Lai et al. (2010)	Present Behavioral Examination	✓				✓				
Léger and Banks (2014)	NPI-Q			✓	✓		✓	✓	✓	✓
Liu et al. (2020)	NPI-Q							✓	✓	
Murray et al. (2014)	Psychiatric evaluation							✓	✓	
Nelson et al. (2023)	NPI-Q			✓	✓		✓	✓	✓	✓
Pillai et al. (2022)	NPI-Q				✓ (844)		✓ (1,025)	✓ (714)	✓	
Scarioni et al. (2020)	Medical records				✓			✓	✓	
Sennik et al. (2016)	NPI-Q					✓ (813)	✓ (813)	✓ (257)	✓ (257)	
Shaw et al. (2024)	NPI-Q			✓ (595)	✓ (201)		✓ (115)			
Sweet et al. (2000)	BEHAVE-AD, psychiatric interview							✓	✓	
Tekin et al. (2001)	NPI-Q			✓ (13)	✓ (10)		✓ (24)	✓ (9)	✓ (14)	✓ (14)
Tsuang et al. (2009)	Neuropsychological evaluation			✓ (73)	✓ (20)	✓ (90)	✓ (90)	✓ (67)	✓ (27)	✓ (83)
Victoroff et al. (1996)	BEHAVE-AD, RAGS					✓	✓			
Vik-Mo et al. (2019)	Norwegian NPI-Q							✓ (20)	✓ (18)	
Zubenko et al. (1991)	Neurological/psychiatric interview							✓	✓	
**References**	**Behavioral assessment tool**	**Hyperactivity**	**Impulsivity**	**Irritability**	**Disinhibition**	**Aggressivity**	**Agitation**	**Delusions**	**Hallucinations**	**Aberrant motor behavior**
Asaoka et al. (2010)	*N/A*	✓		✓			✓	✓	✓	
Beck et al. (2022)	NPI-Q			✓	✓	✓	✓			✓
Fujishiro et al. (2015)	Anamnesis							✓		
Hirano et al. (2020)	*N/A*							✓	✓	
Iwasaki et al. (2018)	Anamnesis	✓	✓	✓	✓	✓	✓			✓
Kawakami et al. (2023)	*N/A*				✓	✓		✓	✓	
Shibuya-Tayoshi et al. (2005)	*N/A*			✓	✓		✓			
Tartaglia et al. (2008)	*N/A*	✓	✓	✓			✓		✓	✓
Asaoka et al. (2010)	Neuropsychological evaluation		✓		✓			✓	✓	

✓, Condition satisfied; n, number of patients; *N*, data not available; NPI-Q, Neuropsychiatric Inventory Questionnaire; MOUSEPAD, Manchester and Oxford Universities Scale for the Psychopathological Assessment of Dementia; BEHAVE-AD, Behavioral Pathology in Alzheimer's Disease Rating Scale; RAGS, Relatives' Assessment of Global Symptomatology.

### 3.6 Neuropathological characteristics

The neuropathological characterization of the entire sample revealed the following definite diagnoses: 14,417 cases (76.92%) were diagnosed with AD; 4,393 (23.44%) with DLB; 1,495 (7.98%) with FTLD; 614 (3.28%) with Limbic-predominant Age-related TDP-43 Encephalopathy Neuropathological Change (LATE-NC); 3,681 (19.64%) with Cerebral Amyloid Angiopathy (CAA); and 4,493 (23.97%) with Vascular Dementia (VaD - Table 4). Most of the studies, specifically 20 out of 29, reported the presence of multiple pathologies. Notably, 15 of these studies identified the coexistence of Alzheimer's Disease Neuropathological Change (ADNC) and Lewy-Related Pathology (LRP), including incidental Lewy Body Disease cases. ADNC was the predominant and most extensively characterized pathology. According to the Montine classification (low-intermediate-high) and Braak stages (I-VI), the severity of ADNC was determined based on the distribution of beta-amyloid plaques and phosphorylated tau (pTAU) deposits (Montine et al., 2012). Of the 11,486 cases with ADNC severity available, 1,794 (15.62%) were classified as low AD, corresponding to a Braak's stage I-II, while 9,691 cases (84.37%) were categorized as intermediate/high AD, corresponding to a Braak's stage III-VI. In the remaining cases, the severity of the pathology was either unspecified or could not be precisely determined (Table 4). Among the 1,495 cases with FTLD where data were available, the underlying proteinopathy was identified as tauopathy in 567 cases (37.93%), TAR DNA-binding protein 43 (TDP-43) proteinopathy in 248 cases (16.59%), and Fused in Sarcoma (FUS) pathology in 8 cases (0.54%; Table 4). Among case reports and case series, the primary neuropathological diagnosis was AD in 3 subjects, atypical Primary Age-Related Tauopathy (PART) in 2 subjects, FTLD due to Ubiquitin and TDP-43 proteinopathy in 1 case, Argyrophilic Grains Disease (AGD) in 1 case, Globular Glial Tauopathy Type I in 1 subject, and localized amygdala degeneration in the remaining case (Table 4). Of the 3 subjects with a primary diagnosis of AD, 2 were classified as high AD and 1 as intermediate AD. Co-occurrence of proteinopathies was identified in 4 out of 9 cases, with amygdala stage III AGD associated with 2 AD cases, one of which also presented with amygdala-predominant LRP. Additionally, mild AD was reported in conjunction with Globular Glial Tauopathy Type 1, and LATE-NC was found alongside AGD in 1 case. The significant proteinopathy burden in the amygdala was emphasized in 4 articles, 3 documenting the coexistence of multiple proteinopathies. In summary, the data available in the literature indicate that the severity of tauopathy and the presence of multiple proteinopathies are the factors most strongly associated with HIDA-P disorders. From a topographical perspective, the amygdala appears to play a central role in the development of these disorders.

**Table 4:** Summary of the neuropathological characteristics the articles included.

**Observational studies**						
**References**	**ADNC, n (low AD, n; int./high AD, n)**	**DLB, n**	**FTLD, n (FTLD-tau, n; FTLD-TDP, n; FTLD-FUS, n)**	**LATE-NC, n**	**Vascular pathology, n**	**CAA, n**
Ballard et al. (1999)	40	40	0	0	0	0
Borges et al. (2019)	765	*N/A*	122	*N/A*	*N/A*	*N/A*
Devanand et al. (2022)	1,246	658	303	*N/A*	568	1,117
Echávarri et al. (2013)	40 (0; 40)	0	0	*N/A*	40	*N/A*
Ehrenberg et al. (2018)	455 (329; 126)	0	0	0	*N/A*	*N/A*
Esteban de Antonio et al. (2020)	40 (0; 40)	17	*N/A*	32	30	*N/A*
Farber et al. (2000)	109	39	0	*N/A*	*N/A*	*N/A*
Ferman et al. (2013)	84 (3; 81)	41	0	0	0	0
Förstl et al. (1994)	56	7	*N/A*	*N/A*	*N/A*	*N/A*
Garcia-Segura et al. (2019)	2,959 (831; 2,128)	*N/A*	0	0	0	0
Gauthreaux et al. (2022)	1,100 (185; 915)	457	0	365	*N/A*	*N/A*
Gibson et al. (2023)	217 (0; 217)	88	64	*N/A*	184	45
Harding et al. (2002)	1	29	*N/A*	*N/A*	*N/A*	*N/A*
Hirsch-Reinshagen et al. (2023)	11 (4; 7)	1	57	*N/A*	1	*N/A*
Lai et al. (2010)	22 (*N*; 21)	*N/A*	*N/A*	*N/A*	3	*N/A*
Léger and Banks (2014)	578	0	110 (33; 0; 0)	*N/A*	*N/A*	*N/A*
Liu et al. (2020)	117	0	0	53	0	*N/A*
Murray et al. (2014)	26 (0; 26)	15	*N/A*	*N/A*	*N/A*	*N/A*
Nelson et al. (2023)	167 (0; 167)	140	0	164	169	75
Pillai et al. (2022)	2,091 (0; 2,091)	1,235	*N/A*	*N/A*	2,402	*N/A*
Scarioni et al. (2020)	35 (0; 35)	3	146 (42; 47; 8)	*N/A*	*N/A*	*N/A*
Sennik et al. (2016)	1,716 (0; 1,716)	*N/A*	*N/A*	*N/A*	332	*N/A*
Shaw et al. (2024)	2,249 (438; 1,811)	1,521	693 (492; 201; 0)	*N/A*	738	2,420
Sweet et al. (2000)	24	11	*N/A*	*N/A*	*N/A*	*N/A*
Tekin et al. (2001)	31 (0; 31)	0	0	*N/A*	*N/A*	*N/A*
Tsuang et al. (2009)	95 (0; 95)	75	*N/A*	*N/A*	*N/A*	*N/A*
Victoroff et al. (1996)	99	0	0	0	*N/A*	0
Vik-Mo et al. (2019)	31 (4; 27)	16	*N/A*	*N/A*	26	24
Zubenko et al. (1991)	13	*N/A*	*N/A*	*N/A*	*N/A*	*N/A*
**Case reports and case series**								
**References**	**Neuropathological diagnosis**	**ADNC (Braak stage)**	**LRP**	**FTLD**	**LATE-NC**	**Vascular pathology**	**CAA**	**AGD**
Asaoka et al. (2010)	AGD with LATE-NC	✓ (Braak II)			✓			✓
Beck et al. (2022)	Intermediate AD	✓ (Braak IV)	✓					✓
Fujishiro et al. (2015)	High AD	✓ (Braak V)						
Hirano et al. (2020)	GGT type 1	✓ (Braak II)				✓	✓	
Iwasaki et al. (2018)	High AD	✓ (Braak VI)					✓	✓
Kawakami et al. (2023)	Atypical PART pathology							
Shibuya-Tayoshi et al. (2005)	Localized amygdala degeneration	✓ (Braak I)						✓
Tartaglia et al. (2008)	FTLD-TDP			✓		✓		

n, number of subjects; *N/A*, data not available; ADNC, Alzheimer's Disease Neuropathological Changes; DLB, Dementia with Lewy Bodies; FTLD, Fronto-Temporal Lobar Degeneration; LATE-NC, Limbic-predominant Age-related; TDP-43, Encephalopathy Neuropathological Change; CAA, Cerebral Amyloid Angiopathy; LRP, Lewy-Related Pathology; AGD, Argyrophilic Grain Disease; GGT, Globular Glial Tauopathy; PART, Primary Age-Related Tauopathy.

## 4 Discussion

Given the high prevalence of BPSD throughout the course of dementia, a more comprehensive understanding of their pathophysiology is crucial for developing more effective strategies to manage and treat neuropsychiatric symptoms. This is particularly relevant for the HIDA-P domains, which are associated with unfavorable prognostic implications, have the greatest clinical and healthcare impact, and impose a significant burden on caregivers (Lyketsos et al., 2002; Cerejeira et al., 2012). Indeed, this systematic review provides a detailed analysis of the neuropathological characteristics associated with the HIDA-P domains of BPSD. This topic is both highly relevant and timely, as understanding these neuropathological changes could guide future research efforts to better clarify the underlying pathophysiology of these disorders. The main issues are addressed below, following the order of the results, focusing specifically on the quality and limitations of the articles, the socio-demographic characteristics of the population studied, their clinical features, and the key neuropathological findings.

### 4.1 Quality and limitations of the articles

All the reviewed articles are of moderate to high quality, with a predominance of high-quality studies, particularly case-control and cohort studies, as well as case reports. This reflects the significant interest generated by the topic and the accuracy of the clinical-neuropathological correlations. As a result, no articles were excluded due to a low score in the quality assessment. However, due to the considerable heterogeneity among the included studies, conducting a meta-analysis was not feasible; therefore, a narrative synthesis approach was adopted to summarize the findings. While this method allows for the inclusion of different study designs, it also has limitations, such as the inability to perform statistical analyses to quantify overall effects or heterogeneity, and the potential for interpretative bias. These limitations may influence the robustness of the conclusions, and the results should be interpreted with caution. Additionally, findings from case reports and case series should be approached carefully, as their generalizability is limited. These reports typically focus on unique clinical and neuropathological cases with HIDA-P symptoms that may not reflect the broader population. Nonetheless, these studies provide valuable insights into individual behavioral trajectories, which are frequently overlooked in larger case series. Despite their heterogeneity and limited number, all the studies analyzed are methodologically robust and provide neuropathological data that reflect the current standards in diagnostic practice. They offer a compelling overview of the underlying neuropathological alterations associated with the HIDA-P cluster. Moreover, the relatively recent median year of publication suggests the ongoing relevance of this topic in current research. Most of the studies were conducted in North America, while a significant portion of case reports originated from Japan providing a regional perspective. The predominance of studies from the United States of America may reflect the relatively easier access to brain tissue, supported by networks such as the National Alzheimer's Coordinating Center, which coordinates data distribution from multiple brain banks (Danner et al., 2024). However, the lack of data from other geographical and cultural contexts limits the ability to assess potential differences in proteinopathies and the prevalence of HIDA-P disorders across various ethnic populations. Cultural factors may influence both the manifestation and interpretation of HIDA-P symptoms, as well as the impact of social and physical environments, particularly in less supportive settings. Variations in healthcare access and cultural awareness of dementia can affect the recognition, diagnosis, and management of BPSD. This highlights the necessity for more comprehensive and inclusive research conducted in diverse geographic locations to mitigate potential biases and enhance the generalizability of findings.

### 4.2 Demographic trends

The demographic data indicate no differences in gender distribution regarding the prevalence of HIDA-P domains. A recent study by Lee et al. (2017) in a Korean cohort of AD patients found no gender differences in the HIDA-P domains. Conversely, a study by Lövheim et al. (2009) on sex differences in BPSD found a significant prevalence of aggressive and inappropriate behaviors in male patients, with no observed difference in hallucinations. Most subjects had a mean age at dementia onset in their 70s, with an average disease duration of approximately 7 years. The relatively early age of onset may be attributable to the inclusion of individuals diagnosed with FTD, which typically occurs in people in their 50s and 60s, as well as some type of AD (i.e., early-onset Alzheimer's disease). Moreover, the participants had a relatively high level of education, with an average of 14 years, suggesting that the development of HIDA-P symptoms may not be directly influenced by cognitive reserve. However, it is also plausible that individuals with higher educational backgrounds are more likely to participate in brain donation programs.

### 4.3 Clinical diagnosis and relationship between behavior and cognition

AD was the predominant clinical diagnosis, confirming its role as the most common form of neurodegenerative dementia (World Health Organization, 2024). However, it is important to recognize that AD encompasses multiple clinical variants (Avelar-Pereira et al., 2023). Additionally, under the diagnostic label of “Alzheimer's Disease,” other forms of dementia may be concealed, only identifiable through neuropathological examination. Several studies already suspected the presence of mixed dementia during the clinical diagnostic process, underscoring that this condition may be more prominent than previously recognized (Sennik et al., 2016; Victoroff et al., 1996; Echávarri et al., 2013; Esteban de Antonio et al., 2020; Gauthreaux et al., 2022). Indeed, the Rush Memory and Aging Project demonstrated that among the 94% of participants with at least one known neuropathology, 78% had two or more, 58% had three or more, and 35% had four or more (Bennett et al., 2012). These data emphasize the role of neuropathology in refining clinical diagnoses. The review highlights NPI-Q as the most widely used and validated tool for assessing behavioral symptoms during dementia. However, its retrospective approach, relying on caregiver reports, introduces potential limitations, such as recall bias and reduced accuracy in symptom reporting (Kaufer et al., 2000). Despite these drawbacks, it remains a cornerstone in the evaluation of neuropsychiatric symptoms due to its established validity and widespread acceptance in dementia (Pillai et al., 2022). Regarding the results of the neuropsychological characteristics, agitation and delusions emerged as the most prevalent HIDA-P symptoms, affecting approximately 11% and 14% of cases, respectively. These were followed by hallucinations, disinhibition and irritability, which were observed in around 7% of cases. The higher frequency of agitation may reflect its broad definitions, which encompass diverse manifestations across different contexts, warranting more precise characterization. Concerning the relationship between BPSD and cognitive decline, the findings of Pillai et al. indicate that the initial cognitive profile may predict specific HIDA-P symptoms. For instance, early executive dysfunction was linked to a higher risk of developing delusions, disinhibition, visual hallucinations, and personality changes, while early visuospatial symptoms were associated with an increased risk of visual hallucinations (Pillai et al., 2022). These results may reflect that disruptions in specific neural circuits contribute to the manifestation of specific HIDA-P symptoms, along with distinct patterns of cognitive impairment. Based on global cognition assessed via MMSE, moderate dementia appears to reflect the overall cognitive status of the population with HIDA-P symptoms. These findings suggest that such symptoms typically emerge during an intermediate disease phase, when affected circuits are impaired but not yet completely degraded. Despite that, behavioral symptoms can occasionally precede the formal diagnosis of dementia. For instance, MBI describes an early clinical state in which subtle behavioral changes signal incipient cognitive decline, potentially indicating the emergence neurodegenerative processes (Ismail et al., 2016). Although these symptoms are generally mild, frequently identified as personality changes, they may reflect the early involvement of neural circuits essential for behavioral regulation. On the other hand, when the HIDA-P symptoms appear early during the course of dementia, they often indicate an unfavorable prognosis (Cerejeira et al., 2012; Farber et al., 2000; Sweet et al., 2000). To confirm this, the study conducted by Scarmeas et al. demonstrated that disruptive behaviors in patients with early-stage Alzheimer's disease, such as verbal outbursts, physical threats or violence, and agitation or restlessness, were associated with an increased risk of cognitive and functional decline, as well as a higher likelihood of institutionalization (Scarmeas et al., 2007). Moreover, the risk of developing hallucinations and delusions progressively increased during the first 3 years of patient observation, and it is also linked to greater cognitive decline (Ropacki and Jeste, 2005). In line with these findings, Sennik et al. reported that the subjects with agitation, regardless of ADNC severity, were associated with worse MMSE scores (Sennik et al., 2016). Conversely, the study by Liu et al. revealed that a more severe cognitive impairment (higher CDR score), which was observed in individuals with mixed dementia (combination of ADNC and LATE-NC), was associated with older age and lower total NPI score and NPI sub-scores in comparison to pure pathologies (ADNC or LATE-NC alone). These findings suggest that a combination of proteinopathies enhances neurodegeneration leading to multiple and deep neural circuit disruption that results in more severe cognitive impairment and behavioral blunting (Liu et al., 2020). As a whole, these observations indicate that HIDA-P symptoms exhibit an “irritative” or “productive” nature, arising from neural dysfunction rather than complete circuit destruction. Other symptoms, such as apathy, represent a behavioral “shutdown,” characterized by emotional blunting and behavioral flattening, indicative of more advanced circuit damage (Esteban de Antonio et al., 2020; Pillai et al., 2022; Nelson et al., 2023; Gibson et al., 2023; Devanand et al., 2022). In essence, HIDA-P symptoms are more prevalent and pronounced during the intermediate stages of the disease, but they may also mark an inflection point toward rapid cognitive and functional deterioration, reflecting more severe neurodegenerative processes, as discussed in the following section.

### 4.4 Neuropathological insights: ADNC, TAU pathology, FTLD, co-occurring proteinopathies and vascular pathology

Certain HIDA-P symptoms are recognized as predictors of specific neuropathological profiles (Shaw et al., 2024). This is outlined in the diagnostic criteria for specific NCDs. There is a well-established correlation between visual hallucinations and DLB, as well as between apathy and disinhibition and FTD, particularly in the bvFTD (Mckeith et al., 2017; Rascovsky et al., 2011). A relationship between specific BPSD and underlying proteinopathies can be hypothesized. Apart from DLB, where the pathology is directly associated with the clinical presentation (as suggested by the term “Lewy bodies”) (Harding et al., 2002), the relationship between BPSD and distinct neuropathologies remains uncertain. As demonstrated by the results of this review and supported by recent findings from Nelson et al., no specific behavioral disorder can be reliably attributed to a single proteinopathy or a defined combination of proteinopathies (Nelson et al., 2023). This is particularly relevant in the context of ADNC. While BPSD associated with DLB and bvFTD tend to manifest as stereotypical clinical patterns, the neuropsychiatric presentations related to the AD spectrum are highly heterogeneous (Gibson et al., 2023; Shaw et al., 2024; Harding et al., 2002; Borges et al., 2019; Devanand et al., 2022; Ballard et al., 1999; Ferman et al., 2013). AD is increasingly recognized as a heterogeneous condition with significant variability in both clinical phenotypes and underlying pathophysiology. Indeed, ADNC is frequently complicated by additional proteinopathies, such as LRP or TDP-43 inclusions, or coexisting vascular pathology. This overlap results in a complex pathological landscape, contributing to diverse behavioral and psychological manifestations. To support this, the neuropathological findings from this review indicate ADNC as the predominant pathology, frequently coexisting with other additional proteinopathies along with vascular pathology and/or CAA. Several studies emphasize that HIDA-P symptoms, particularly agitation, aggressive behavior, and psychotic symptoms are closely associated with underlying ADNC (Sennik et al., 2016; Esteban de Antonio et al., 2020; Pillai et al., 2022; Sweet et al., 2000; Nelson et al., 2023; Gibson et al., 2023; Devanand et al., 2022; Shaw et al., 2024; Léger and Banks, 2014; Förstl et al., 1994). To confirm this significant association, Leger et al. found that psychotic symptoms and agitation were more common in patients clinically misdiagnosed with bvFTD but later confirmed to ADNC, compared to those with definite FTLD pathology (Léger and Banks, 2014).

Further, the present review highlights a higher prevalence of intermediate and advanced ADNC compared to low-grade forms, suggesting the central role of TAUopathy in driven neurodegeneration and the consequent clinical and behavioral manifestations. However, the role of amyloid burden remains less clear. While the extent and distribution of TAU pathology show a positive correlation with the presence of HIDA-P symptoms (Murray et al., 2014; Farber et al., 2000; Gibson et al., 2023; Förstl et al., 1994; Lai et al., 2010; Zubenko et al., 1991; Tekin et al., 2001; Ehrenberg et al., 2018), no significant increase in beta-amyloid burden or senile plaques has been reported in relation to the presence or severity of HIDA-P manifestations (Farber et al., 2000; Sweet et al., 2000; Förstl et al., 1994; Lai et al., 2010; Tekin et al., 2001). An exception is the study by Zubenko et al., which identified an association between psychosis and increased severity of senile plaques, though this correlation was confined specifically to the pre-subiculum area (Zubenko et al., 1991). These findings confirm the major role of TAU pathology in disrupting brain circuits involved in developing HIDA-P symptoms, with amyloid pathology playing a secondary role. Histologically, TAU pathology is characterized by intracellular neurofibrillary tangles (NFTs) and neuropil threads composed of hyperphosphorylated TAU protein (pTAU). During the course of AD, the progression of pTAU deposition follows a distinct topographical pattern: initially confined to the entorhinal cortex, it extends to the hippocampus, limbic structures, temporo-parietal regions, and eventually the entire neocortex, with variations dependent on individual patient characteristics (Braak et al., 2003; Braak and Braak, 1991; Vogel et al., 2021). The studies reviewed emphasize the importance of specific brain regions, particularly limbic areas such as the hippocampus, parahippocampal gyrus, orbitofrontal cortex, and anterior cingulate cortex, where the accumulation of pTAU leads to the development of HIDA-P symptoms (Förstl et al., 1994; Lai et al., 2010; Tekin et al., 2001). As Tekin and Cummings (2002) noted, dysfunction in limbic regions impairs emotional regulation, leading to heightened emotional reactivity, impulsivity, and aggressive behaviors (Tekin and Cummings, 2002). For instance, Lai et al. found that increased hippocampal NFTs are associated with more severe aggressive behaviors and chronic aggression. Additionally, the aggression factor score, determined by the sum of the highest scores during follow-up, was identified as a strong predictor of NFT severity (Lai et al., 2010). Alongside the study of Lai et al. (2010) and Förstl et al. (1994) identified a significant correlation between delusions in AD patients and the pTAU amount specifically in the parahippocampal region. Among lobar limbic structures, the orbitofrontal cortex plays a central role in regulating emotions, decision-making, and impulse control by integrating sensory information and socio-emotional cues. The orbitofrontal circuit connects the frontal monitoring systems to the subcortical components of the limbic system. Disruption of this circuit impairs the brain's ability to properly evaluate and respond to emotional stimuli, often leading to increased irritability, impulsivity, and agitation (Tekin and Cummings, 2002). Tekin et al. (2001) identified a significant association between agitation and NFT load in the orbitofrontal cortex and left anterior cingulate; Murray et al. (2014) obtained similar results. However, not all studies reach the same conclusions. For instance, Farber et al. (2000) present results that are not completely in line with those reported in the other studies. Notably, they found that psychosis was associated with a greater density of neocortical NFTs, distributed more widely across the brain. This suggests a more extensive neocortical involvement in the development of psychosis in individuals with AD, rather than a focus on the hippocampus or entorhinal cortex (Farber et al., 2000). Nonetheless, this study considered only pure psychotic symptoms, such as hallucinations and delusions, and not the entire HIDA-P spectrum. Eventually, the data indicate that the localization of TAU pathology within the limbic system and frontal lobe, particularly the anterior cingulate and orbitofrontal cortex, plays a major role in the emergence of HIDA-P symptoms.

Similar to ADNC, the various proteinopathies underlying FTLD, especially TAU and TDP-43 pathology, primarily target the fronto-limbic regions. Impulsivity and disinhibition, which belong to the HIDA-P cluster and they are hallmark symptoms of bvFTD, stem from dysfunctions in the anterior cingulate and orbitofrontal cortices, impairing the processing of socio-emotional information (Coccaro et al., 2011). This is also consistent with the evidence showing that the distribution of TDP-43 pathology in bvFTD initially targets regions crucial for emotional processing, such as the amygdala, orbital gyri, and gyrus rectus (Josephs et al., 2014). Scarioni et al. (2020) examined neuropsychiatric symptoms across different FTLD histotypes and found no substantial differences in HIDA-P symptoms between FTLD-TDP, FTLD-TAU, FTLD-FUS, and non-FTLD cases (mainly frontal variants of AD). However, certain clinical peculiarities suggest specific underlying neuropathologies: (1) oral disinhibition (hyperorality) is more characteristic of FTLD than non-FTLD; (2) hallucinations are suggestive of FTLD-TDP (not limited to C9orf72 mutations); and (3) perseverative or compulsive behaviors are more frequent in FTLD-TDP type B or C. Moreover, Scarioni et al. found ADNC co-occurring in nearly half of FTLD-TDP cases, highlighting the relevance of mixed pathologies affecting the limbic system (Scarioni et al., 2020). Beyond its role in FTLD, TDP-43 proteinopathy also constitutes the neuropathological signature of LATE-NC, an amnestic syndrome in older adults which follows a stereotyped pattern of protein accumulation from the amygdala to the hippocampus and the middle frontal gyrus. LATE-NC often co-occurs with other neurodegenerative diseases, particularly ADNC (Nelson et al., 2019). Several studies have investigated the relationship between HIDA-P symptoms and LATE-NC, either alone or in combination with other proteinopathies such as ADNC. Gauthreaux et al. (2022) found a prevalence of apathy, disinhibition, agitation, and personality changes in cases with low to intermediate ADNC combined with LATE-NC. Similarly, Sennik et al. (2016) reported an increased risk of agitation and aggression in patients with both ADNC and TDP-43 proteinopathy, compared to those with ADNC alone (Gauthreaux et al., 2022). However, in cases with severe ADNC co-occurring with LATE-NC, worse cognitive decline predominated without significant behavioral manifestations (Gauthreaux et al., 2022). Liu et al. found that patients with co-occurring ADNC and LATE-NC had lower total NPI-Q score, particularly in the “agitation” domain (encompassing the whole HIDA spectrum), compared to those with pure ADNC. Additionally, the “frontal” factor scores (including elation, apathy, disinhibition, and irritability) were lower in the group with both ADNC and LATE-NC than in cases with pure LATE-NC (Liu et al., 2020). These results suggest that the presence of concomitant LATE-NC in ADNC does not necessarily correlate with a greater burden of neuropsychiatric symptoms, which can vary in relation to the severity of the underlying pathology. As a matter of fact, the discrepancy in these findings may be explained by the absence of comprehensive neuropathological data in Liu et al.'s study, such as the precise stages of LATE-NC, while the severity of ADNC can be inferred as moderate to high AD. It is possible that an advanced stage of both pathologies, associated with widespread protein deposits, may disrupt neuronal circuits to a degree that leads to a reduction in behavioral symptoms rather than an exacerbation.

The limbic involvement in LRP appears crucial, particularly in the case of DLB. Ferman et al. (2013) found that the occurrence of visual hallucinations (VHs) in DLB correlates with a higher presence of Lewy bodies in the limbic system. Further, they noted that if VHs occurred within the first 5 years of dementia onset, a DLB diagnosis is more likely than AD (Ferman et al., 2013). However, the generation of VHs involves multiple cortical regions, including the occipital cortex, indicating that their association with the limbic system is not exclusive. Supporting this view, Tsuang et al. observed that neocortical LRP is more commonly associated with VHs than limbic involvement alone. Further, the study showed that ADNC associated with concomitant LRP was the most frequent neuropathological finding in patients with VHs, although the prevalence of this co-occurrence did not significantly differ between those with and without VHs (74% vs. 62%) (Tsuang et al., 2009). Similarly, Devanand et al. (2022) highlighted that as LRP progresses from the brainstem to the limbic system and neocortex, VHs tend to increase in frequency. This further emphasizes that mixed pathologies, particularly LRP and ADNC, are strongly linked to the presence and severity of VHs. These findings remark the importance of multiple neuropathologies in driving behavioral disturbances within the HIDA-P cluster. Beyond the relationship between LRP and VHs, a strong association emerged between the co-existence of ADNC and LRP in driving agitation and aggression (Devanand et al., 2022), as well as a higher burden of BPSD (Gibson et al., 2023). The presence of multiple proteinopathies tends to exacerbate behavioral disturbances more than pure pathologies, suggesting an additive rather than synergistic effect (Gibson et al., 2023). However, other studies found no clear relationship between mixed pathologies and HIDA-P symptoms (Esteban de Antonio et al., 2020; Liu et al., 2020; Tsuang et al., 2009). This inconsistency may reflect the distribution patterns of individual proteinopathies.

Eventually, Nelson et al. further reported that a greater burden of proteinopathies, known as the “quadruple misfolding proteinopathy” (ADNC, neocortical LBs, and LATE-NC) correlates with an increased prevalence and complexity of BPSD, including symptoms within the HIDA-P clusters (Nelson et al., 2023). Esteban de Antonio et al. (2020) proposed that regional rather than diffuse involvement of pathologies may explain the occurrence of HIDA-P symptoms. This concept aligns with the severity of proteinopathies: higher pathological burdens correspond to more widespread brain involvement, as described by several neuropathological staging systems (McAleese et al., 2017). The study further suggests that the spreading of pathologies throughout the brain, and the consequent greater neuropathological burden, may alleviate HIDA-P symptoms, with apathy being the exception (Esteban de Antonio et al., 2020). This observation aligns with the hypothesis proposed by Cummings (1985), which suggest that a certain level of pathology is necessary for the development of psychotic symptoms, while a degree of cognitive preservation is essential for contextualizing these symptoms (Förstl et al., 1994; Cummings, 1985). Indeed, several studies have demonstrated that the preservation of neuronal counts in key limbic regions correlates with the manifestation of HIDA-P symptoms (Victoroff et al., 1996; Förstl et al., 1994). In summary, the accumulation of proteinopathies within specific brain regions appears to disrupt key circuits involved in the development of HIDA-P symptoms, triggering their manifestation during dementia's clinical progression. This process highlights the interplay between neuropathological burden and symptomatology, where topographical involvement and partial neuronal preservation contribute to the timing and complexity of these symptoms.

Concerning the vascular impact on behavior, several studies have been conducted to investigate the relationship between vascular lesions and BPSD (Ballard et al., 2000; Aharon-Peretz et al., 2000; Sultzer et al., 1993; Staekenborg et al., 2010). A notable limitation in these studies is the absence of neuropathological confirmation. The findings indicate a higher prevalence of affective symptoms, such as depression and apathy, in patients with VaD compared to those with AD, where psychotic symptoms are more frequently observed. Although only a few studies include neuropathological validation of vascular changes, those that do confirm previous clinical findings. Notably, they emphasize the association between vascular lesions and inhibitory behaviors (Echávarri et al., 2013; Esteban de Antonio et al., 2020; Devanand et al., 2022). In contrast, aggressive and agitated behaviors, characteristic of the HIDA cluster, tend to be less prominent in the presence of vascular lesions (Sennik et al., 2016). The study by Echávarri et al. (2013) reported a slightly elevated prevalence of depression in patients with VaD relative to those with AD. However, no consistent differences were observed between the two groups regarding the presence of HIDA-P symptoms (Echávarri et al., 2013). Vik-Mo et al. (2019) reported that patients with CAA comorbid with AD, but not those with small vessel disease, exhibited higher mean NPI item scores for delusions and hallucinations. These findings suggest that vascular lesions may disrupt neural circuits responsible for regulating actions and behaviors, impairing their normal function (Kalaria, 2016). This disruption appears to lead to a suppression of more complex and “productive” behavioral symptoms (i.e., HIDA-P symptoms), resulting instead in a predominance of apathy. Therefore, the frequent presence of cerebrovascular lesions in conjunction with proteinopathies, may contribute to complicate the phenotypic presentation.

### 4.5 What we learn from the “case reports:” the role of the amygdala

The systematic review of the case reports on the neuropathology of the HIDA-P domains of BPSD reveals complex interactions between neurodegenerative processes and the presentation of neuropsychiatric symptoms. The study of individual cases, even if anecdotal, enables detailed tracking of clinical trajectories and the sequential manifestation of symptoms, allowing the analysis of their evolution and clustering. Furthermore, it facilitates the precise neuropathological characterization of individual patients, accounting for their unique features. Such detailed qualitative information is often lost in studies involving large cohorts. In alignment with the hypothesis proposed by several cited authors, case reports and case series emphasize the significance of co-occurring proteinopathies and the involvement of specific limbic structures in the manifestation of hyperactivity, impulsivity, disinhibition, aggression, and psychosis (Esteban de Antonio et al., 2020; Nelson et al., 2023; Gibson et al., 2023). A consistent finding across these cases is the pivotal role of the amygdala, which appears implicated in almost all instances (Shibuya-Tayoshi et al., 2005; Asaoka et al., 2010; Beck et al., 2022; Iwasaki et al., 2018). The amygdala emerges as the primary emotional hub within the limbic system, significantly influencing symptom onset and severity. Widely recognized for its crucial role in emotional regulation, the amygdala is vulnerable to structural alterations, particularly volume reduction, along with connectivity disruptions (i.e., dysconnectivity between the amygdala and frontal regions). These alterations are thought to contribute to the development of psychotic symptoms (Delavari et al., 2023). Further, the disruption of functional connections with the frontal and orbitofrontal cortices, appears to play a crucial role in impairing control over socio-emotional information processing and decision-making, resulting in disinhibition and impulsivity (Casanova et al., 2011). To confirm the amygdala and its networks as key regions involved in the origin of HIDA-P symptoms, preliminary evidence from studies on BPSD in early AD suggests that the accumulation of NFTs in these areas may be associated with loss of motivation, social inappropriateness, and agitation (Stouffer et al., 2024). Evidence suggests that disruption of amygdala circuitry, caused by the deposition of multiple misfolded proteins, is a critical factor in accelerating the progression of neurodegenerative diseases, impacting both cognitive decline and neurobehavioral impairment (Nelson et al., 2018). These findings remark the significance of localized regional alterations, rather than diffuse changes, in the development of BPSD (Esteban de Antonio et al., 2020; Casanova et al., 2011). In addition to the central role of the amygdala, age-related limbic TAUopathies, such as Argyrophilic Grain Disease (AGD), are frequently identified as co-pathologies in case reports (Shibuya-Tayoshi et al., 2005; Asaoka et al., 2010; Beck et al., 2022; Iwasaki et al., 2018). According to Ferrer et al., AGD is a sporadic neurodegenerative disorder typically observed in the elderly. It is characterized by the presence of argyrophilic grains and hyperphosphorylated 4R tau pre-tangle neurons within the limbic system, contributing to approximately 5% of all cases of dementia (Ferrer et al., 2008). Further investigation is warranted to elucidate the role of limbic AGD in the development of behavioral abnormalities among the elderly.

## 5 Conclusions

Behavioral disorders are not merely an epiphenomenon arising from cognitive deficits or general dysfunction of the cerebral cortex, nor premorbid personality disorder; rather, they originate from the dysfunction of specific neural circuits and brain regions. In the context of the HIDA-P cluster, the limbic system is notably the most affected, likely due to its role as a convergence point for multiple neurodegenerative processes. The extent and severity of neurodegeneration in these structures are pivotal in determining the clinical expression of symptoms. The emergence of HIDA-P symptoms often indicates a progression of the underlying pathology and may signify a critical turning point during dementia. The intensity of TAU pathology exhibits the strongest correlation with behavioral symptoms; however, the presence of multiple pathologies tends to correlate with increased severity of these symptoms. Indeed, co-pathologies (including ADNC and LRP and/or TDP-43 pathology) are indicative of more complex and potentially severe neurodegenerative phenomena. Conversely, when neurodegenerative processes reach advanced stages, particularly in the presence of vascular damage, the integrity of neural circuits may be compromised or severely impaired. In such cases, HIDA-P symptoms may diminish or resolve, often replaced by symptoms such as apathy and behavioral inhibition. While the same HIDA-P symptom may be associated with different proteinopathies, conversely, a single proteinopathy can exhibit multiple HIDA-P manifestations depending on its localization. Therefore, the topographical characteristics of the lesions appear to play a more significant role than their molecular composition in the emergence of behavioral symptoms. Future research should focus on elucidating the roles of distinct limbic circuits, multiple proteinopathies, and neuroinflammation, to develop new neuropathological classifications. This would enhance the understanding and definition of behavioral disorders, ultimately improving differential diagnosis and patient management, as well as informing more targeted therapeutic strategies.

## Data Availability

The datasets presented in this study can be found in online repositories. The names of the repository/repositories and accession number(s) can be found in the article/Supplementary material.

## References

[B1] Aharon-Peretz J. Kliot D. Tomer R. (2000). Behavioral differences between white matter lacunar dementia and Alzheimer's disease: a comparison on the neuropsychiatric inventory. Dement. Geriatr. Cogn. Disord. 11, 294–298. 10.1159/000017252 10940681

[B2] American Psychiatric Association (2013). Diagnostic and Statistical Manual of Mental Disorders. 5th ed. American Psychiatric Association, editor. Arlington (VA). 10.1176/appi.books.9780890425596

[B3] Asaoka T. Tsuchiya K. Fujishiro H. Arai T. Hasegawa M. Akiyama H. . (2010). Argyrophilic grain disease with delusions and hallucinations: a pathological study. Psychogeriatrics.10, 69–76. 10.1111/j.1479-8301.2010.00318.x 20738810

[B4] Avelar-Pereira B. Belloy M. E. O'Hara R. Hosseini S. M. H. (2023). Decoding the heterogeneity of Alzheimer's disease diagnosis and progression using multilayer networks. Mol. Psychiatry. 28, 2423–2432. 10.1038/s41380-022-01886-z 36539525 PMC10279806

[B5] Ballard C. Holmes C. McKeith I. Neill D. O'Brien J. Cairns N. . (1999). Psychiatric morbidity in dementia with lewy bodies: a prospective clinical and neuropathological comparative study with Alzheimer's disease. Am. J. Psychiat. 156, 1039–1045. 10.1176/ajp.156.7.1039 10401449

[B6] Ballard C. Neill D. O'Brien J. McKeith I. G. Ince P. Perry R. . (2000). Anxiety, depression and psychosis in vascular dementia: prevalence and associations. J. Affect. Disord. 59, 97–106. 10.1016/S0165-0327(99)00057-9 10837878

[B7] Beck G. Shigenobu K. Ukon K. Yamashita R. Yonenobu Y. Morii E. . (2022). An autopsy case of Alzheimer's disease with amygdala-predominant lewy pathology presenting with frontotemporal dementia-like psychiatric symptoms. Neuropathology 42, 147–154. 10.1111/neup.12786 35112739

[B8] Bennett D. A. Schneider J. A. Buchman A. S. Barnes L. L. Boyle P. A. Wilson R. S. (2012). Overview and findings from the rush memory and aging project. Curr. Alzheimer Res. 9, 646–663. 10.2174/156720512801322663 22471867 PMC3439198

[B9] Borges L. G. Rademaker A. W. Bigio E. H. Mesulam M. M. Weintraub S. (2019). Apathy and disinhibition related to neuropathology in amnestic versus behavioral dementias. Am. J. Alzheimers. Dis. Other Demen. 34, 337–343. 10.1177/1533317519853466 31170813 PMC7256964

[B10] Braak H. Braak E. (1991). Neuropathological stageing of Alzheimer-related changes. Acta Neuropathol. 82, 239–259. 10.1007/BF00308809 1759558

[B11] Braak H. Del Tredici K. Rüb U. de Vos R. A. I. Jansen Steur E. N. H. Braak E. . (2003). Staging of brain pathology related to sporadic Parkinson's disease. Neurobiol. Aging 24, 197–211. 10.1016/S0197-4580(02)00065-9 12498954

[B12] Casanova M. F. Starkstein S. E. Jellinger K. A. (2011). Clinicopathological correlates of behavioral and psychological symptoms of dementia. Acta Neuropathol. 122, 117–135. 10.1007/s00401-011-0821-3 21455688

[B13] Cerejeira J. Lagarto L. Mukaetova-Ladinska E. B. (2012). Behavioral and psychological symptoms of dementia. Front. Neurol. 3. 10.3389/fneur.2012.00073 22586419 PMC3345875

[B14] Clinton L. K. Blurton-Jones M. Myczek K. Trojanowski J. Q. LaFerla F. M. (2010). Synergistic interactions between Aβ, Tau, and α-Synuclein: acceleration of neuropathology and cognitive decline. J. Neurosci. 30, 7281–7289. 10.1523/JNEUROSCI.0490-10.2010 20505094 PMC3308018

[B15] Coccaro E. F. Sripada C. S. Yanowitch R. N. Phan K. L. (2011). Corticolimbic function in impulsive aggressive behavior neural systems underlying experience of aggressive impulses. BPS 69, 1153–1159. 10.1016/j.biopsych.2011.02.032 21531387

[B16] Cotta Ramusino M. Imbimbo C. Capelli M. Cabini R. F. Bernini S. Lombardo F. P. . (2024). Role of fronto-limbic circuit in neuropsychiatric symptoms of dementia: clinical evidence from an exploratory study. Front. Psychiat. 15:1231361. 10.3389/fpsyt.2024.1231361 38800068 PMC11119745

[B17] Cummings J. L. (1985). Organic delusions: phenomenology, anatomical correlations, and review. Br. J. Psychiat. 146, 184–197. 10.1192/bjp.146.2.184 3156653

[B18] Danner B. Gonzalez A. D. Corbett W. C. Alhneif M. Etemadmoghadam S. Parker-Garza J. . (2024). Brain banking in the United States and Europe: importance, challenges, and future trends. J. Neuropathol. Exp. Neurol. 83, 219–229. 10.1093/jnen/nlae014 38506125 PMC10951968

[B19] Delavari F. Rafi H. Sandini C. Murray R. J. Latrèche C. Van De Ville D. . (2023). Amygdala subdivisions exhibit aberrant whole-brain functional connectivity in relation to stress intolerance and psychotic symptoms in 22q11.2DS. Transl. Psychiatry. 13:145. 10.1038/s41398-023-02458-7 37142582 PMC10160125

[B20] Devanand D. P. Lee S. Huey E. D. Goldberg T. E. (2022). Associations between neuropsychiatric symptoms and neuropathological diagnoses of Alzheimer disease and related dementias. JAMA Psychiat. 79:359. 10.1001/jamapsychiatry.2021.4363 35171235 PMC8851371

[B21] Echávarri C. Burgmans S. Uylings H. Cuesta M. J. Peralta V. Kamphorst W. . (2013). Neuropsychiatric symptoms in Alzheimer's disease and vascular dementia. J. Alzheimer's Dis. 33, 715–721. 10.3233/JAD-2012-121003 23001706

[B22] Ehrenberg A. J. Suemoto C. K. França Resende E. P. Petersen C. Leite R. E. P. Rodriguez R. D. . (2018). Neuropathologic correlates of psychiatric symptoms in Alzheimer's disease. J. Alzheimers. Dis. 66, 115–126. 10.3233/JAD-180688 30223398 PMC6381997

[B23] Esteban de Antonio E. López-Álvarez J. Rábano A. Agüera-Ortiz L. Sánchez-Soblechero A. Amaya L. . (2020). Pathological correlations of neuropsychiatric symptoms in institutionalized people with dementia. J. Alzheimers. Dis. 78, 1731–1741. 10.3233/JAD-200600 33185596

[B24] Farber N. B. Rubin E. H. Newcomer J. W. Kinscherf D. A. Miller J. P. Morris J. C. . (2000). Increased neocortical neurofibrillary tangle density in subjects with Alzheimer disease and psychosis. Arch. Gen. Psychiatry. 57:1165. 10.1001/archpsyc.57.12.1165 11115331

[B25] Ferman T. J. Arvanitakis Z. Fujishiro H. Duara R. Parfitt F. Purdy M. . (2013). Pathology and temporal onset of visual hallucinations, misperceptions and family misidentification distinguishes dementia with Lewy bodies from Alzheimer's disease. Parkinsonism Relat. Disord. 19, 227–231. 10.1016/j.parkreldis.2012.10.013 23182311 PMC3570751

[B26] Ferrer I. Santpere G. van Leeuwen F. W. (2008). Argyrophilic grain disease. Brain 131, 1416–1432. 10.1093/brain/awm305 18234698

[B27] Finkel S. (2000). Introduction to behavioural and psychological symptoms of dementia (BPSD). Int. J. Geriatr. Psychiatry 15, S2–4. 10.1002/(SICI)1099-1166(200004)15:1+<S2::AID-GPS159>3.0.CO;2-3 10767742

[B28] Fischer C. E. Ismail Z. Schweizer T. A. (2012). Impact of neuropsychiatric symptoms on caregiver burden in patients with Alzheimer's disease. Neurodegener. Dis. Manag. 2, 269–277. 10.2217/nmt.12.19

[B29] Förstl H. Burns A. Levy R. Cairns N. (1994). Neuropathological correlates of psychotic phenomena in confirmed Alzheimer's disease. Br. J. Psychiatry 165, 53–59. 10.1192/bjp.165.1.53 7953058

[B30] Fujishiro H. Iritani S. Hattori M. Sekiguchi H. Matsunaga S. Habuchi C. . (2015). Autopsy-confirmed hippocampal-sparing Alzheimer's disease with delusional jealousy as initial manifestation. Psychogeriatrics 15, 198–203. 10.1111/psyg.12105 25737011

[B31] Gagnier J. J. Kienle G. Altman D. G. Moher D. Sox H. Riley D. . (2013). The CARE guidelines: consensus-based clinical case reporting guideline development. Glob. Adv. Health Med. 2, 38–43. 10.7453/gahmj.2013.008 24416692 PMC3833570

[B32] Garcia-Segura M. E. Fischer C. E. Schweizer T. A. Munoz D. G. (2019). APOE ε4/ε4 is associated with aberrant motor behavior through both lewy body and cerebral amyloid angiopathy pathology in high Alzheimer's disease pathological load. J. Alzheimer's Dis. 72, 1077–1087. 10.3233/JAD-190643 31744003 PMC9680058

[B33] Gauthreaux K. Mock C. Teylan M. A. Culhane J. E. Chen Y. C. Chan K. C. G. . (2022). Symptomatic profile and cognitive performance in autopsy-confirmed limbic-predominant age-related tdp-43 encephalopathy with comorbid Alzheimer disease. J. Neuropathol. Exp. Neurol. 81, 975–987. 10.1093/jnen/nlac093 36264254 PMC9677237

[B34] Gibson L. L. Grinberg L. T. Ffytche D. Leite R. E. P. Rodriguez R. D. Ferretti-Rebustini R. E. L. . (2023). Neuropathological correlates of neuropsychiatric symptoms in dementia. Alzheimer's Dement. 19, 1372–1382. 10.1002/alz.12765 36150075 PMC10033459

[B35] Gilley D. W. Bienias J. L. Wilson R. S. Bennett D. A. Beck T. L. Evans D. A. . (2004). Influence of behavioral symptoms on rates of institutionalization for persons with Alzheimer's disease. Psychol. Med. 34, 1129–1135. 10.1017/S0033291703001831 15554582

[B36] Harding A. J. Broe G. A. Halliday G. M. (2002). Visual hallucinations in Lewy body disease relate to Lewy bodies in the temporal lobe. Brain 125, 391–403. 10.1093/brain/awf033 11844739

[B37] Herrmann N. Lanctôt K. L. Sambrook R. Lesnikova N. Hébert R. McCracken P. . (2006). The contribution of neuropsychiatric symptoms to the cost of dementia care. Int. J. Geriatr. Psychiat. 21, 972–976. 10.1002/gps.1594 16955429

[B38] Hirano M. Iritani S. Fujishiro H. Torii Y. Kawashima K. Sekiguchi H. . (2020). Globular glial tauopathy Type I presenting with behavioral variant frontotemporal dementia. Neuropathology 40, 515–525. 10.1111/neup.12668 33463808

[B39] Hirsch-Reinshagen V. Hercher C. Vila-Rodriguez F. Neumann M. Rademakers R. Honer W. G. . (2023). Psychotic symptoms in frontotemporal dementia with TDP-43 tend to be associated with type B pathology. Neuropathol. Appl. Neurobiol. 49:e12921. 10.1111/nan.12921 37386798 PMC10527970

[B40] Ismail Z. Smith E. E. Geda Y. Sultzer D. Brodaty H. Smith G. . (2016). Neuropsychiatric symptoms as early manifestations of emergent dementia: provisional diagnostic criteria for mild behavioral impairment. Alzheimer's Dement. 12, 195–202. 10.1016/j.jalz.2015.05.017 26096665 PMC4684483

[B41] Iwasaki Y. Deguchi A. Mori K. Ito M. Kawai Y. Mimuro M. . (2018). Pathological diagnosis of combined Alzheimer's disease and argyrophilic grain dementia in a very elderly man who presented with advanced behavioural and psychological symptoms. Psychogeriatrics 18, 421–426. 10.1111/psyg.12342 29993165

[B42] Josephs K. A. Murray M. E. Whitwell J. L. Parisi J. E. Petrucelli L. Jack C. R. . (2014). Staging TDP-43 pathology in Alzheimer's disease. Acta Neuropathol. 127, 441–450. 10.1007/s00401-013-1211-9 24240737 PMC3944799

[B43] Kalaria R. N. (2016). Neuropathological diagnosis of vascular cognitive impairment and vascular dementia with implications for Alzheimer's disease. Acta Neuropathol. 131, 659–685. 10.1007/s00401-016-1571-z 27062261 PMC4835512

[B44] Kaufer D. I. Cummings J. L. Ketchel P. Smith V. MacMillan A. Shelley T. . (2000). Validation of the NPI-Q, a brief clinical form of the neuropsychiatric inventory. J. Neuropsychiatry Clin. Neurosci. 12, 233–239. 10.1176/jnp.12.2.233 11001602

[B45] Kawakami I. Arai T. Ikeda K. Niizato K. Oshima K. Akiyama H. . (2023). Possible association of limbic tau pathology with psychosis or behavioral disturbances: Studies of two autopsied psychiatric patients. Neuropathology 43, 44–50. 10.1111/neup.12870 36341554

[B46] Keszycki R. M. Fisher D. W. Dong H. (2019). The hyperactivity–impulsivity–irritiability–disinhibition–aggression–agitation domain in Alzheimer's disease: current management and future directions. Front. Pharmacol. 10:1109. 10.3389/fphar.2019.01109 31611794 PMC6777414

[B47] Kim B. Noh G. O. Kim K. (2021). Behavioural and psychological symptoms of dementia in patients with Alzheimer's disease and family caregiver burden: a path analysis. BMC Geriatr. 21:160. 10.1186/s12877-021-02109-w 33663416 PMC7934246

[B48] Lai M. K. P. Chen C. P. Hope T. Esiri M. M. (2010). Hippocampal neurofibrillary tangle changes and aggressive behaviour in dementia. Neuroreport 21, 1111–1115. 10.1097/WNR.0b013e3283407204 20890229

[B49] Lee J. Lee K. J. Kim H. (2017). Gender differences in behavioral and psychological symptoms of patients with Alzheimer's disease. Asian J. Psychiatr. 26, 124–128. 10.1016/j.ajp.2017.01.027 28483073

[B50] Léger G. C. Banks S. J. (2014). Neuropsychiatric symptom profile differs based on pathology in patients with clinically diagnosed behavioral variant frontotemporal dementia. Dement. Geriatr. Cogn. Disord. 37, 104–112. 10.1159/000354368 24135712 PMC4041327

[B51] Liu K. Y. Reeves S. McAleese K. E. Attems J. Francis P. Thomas A. . (2020). Neuropsychiatric symptoms in limbic-predominant age-related TDP-43 encephalopathy and Alzheimer's disease. Brain 143, 3842–3849. 10.1093/brain/awaa315 33188391 PMC7805786

[B52] Lo C. K. L. Mertz D. Loeb M. (2014). Newcastle-Ottawa Scale: comparing reviewers' to authors' assessments. BMC Med. Res. Methodol. 14:45. 10.1186/1471-2288-14-45 24690082 PMC4021422

[B53] Lövheim H. Sandman P. O. Karlsson S. Gustafson Y. (2009). Sex differences in the prevalence of behavioral and psychological symptoms of dementia. Int. Psychogeriatr. 21:469. 10.1017/S1041610209008497 19243654

[B54] Lyketsos C. G. Lopez O. Jones B. Fitzpatrick A. L. Breitner J. DeKosky S. . (2002). Prevalence of neuropsychiatric symptoms in dementia and mild cognitive impairment. JAMA 288:1475. 10.1001/jama.288.12.1475 12243634

[B55] McAleese K. E. Walker L. Erskine D. Thomas A. J. McKeith I. G. Attems J. . (2017). TDP-43 pathology in Alzheimer's disease, dementia with Lewy bodies and ageing. Brain Pathol. 27, 472–479. 10.1111/bpa.12424 27495267 PMC8029292

[B56] Mckeith I. G. Boeve B. F. Dickson D. W. Halliday G. Taylor J. Weintraub D. (2017). Diagnosis and management of dementia with Lewy bodies fourth consensus report of the DLB Consortium. Neurology 89, 88–100. 10.1212/WNL.0000000000004058 28592453 PMC5496518

[B57] Moher D. Liberati A. Tetzlaff J. Altman D. G. PRISMA Group. (2009). Preferred reporting items for systematic reviews and meta-analyses: the PRISMA statement. PLoS Med. 6:e1000097. 10.1371/journal.pmed.1000097 19621072 PMC2707599

[B58] Montine T. J. Phelps C. H. Beach T. G. Bigio E. H. Cairns N. J. Dickson D. W. . (2012). National institute on aging–Alzheimer's association guidelines for the neuropathologic assessment of Alzheimer's disease: a practical approach. Acta Neuropathol. 123, 1–11. 10.1007/s00401-011-0910-3 22101365 PMC3268003

[B59] Murray P. S. Kirkwood C. M. Gray M. C. Fish K. N. Ikonomovic M. D. Hamilton R. L. . (2014). Hyperphosphorylated tau is elevated in Alzheimer's disease with psychosis. J. Alzheimer's Dis. 39, 759–773. 10.3233/JAD-131166 24270207 PMC4034758

[B60] Nelson P. T. Abner E. L. Patel E. Anderson S. Wilcock D. M. Kryscio R. J. . (2018). The amygdala as a locus of pathologic misfolding in neurodegenerative diseases. J. Neuropathol. Exp. Neurol. 77, 2–20. 10.1093/jnen/nlx099 29186501 PMC5901077

[B61] Nelson P. T. Dickson D. W. Trojanowski J. Q. Jack C. R. Boyle P. A. Arfanakis K. . (2019). Limbic-predominant age-related TDP-43 encephalopathy (LATE): consensus working group report. Brain 142, 1503–1527. 10.1093/brain/awz099 31039256 PMC6536849

[B62] Nelson R. S. Abner E. L. Jicha G. A. Schmitt F. A. Di J. Wilcock D. M. . (2023). Neurodegenerative pathologies associated with behavioral and psychological symptoms of dementia in a community-based autopsy cohort. Acta Neuropathol. Commun. 11:89. 10.1186/s40478-023-01576-z 37269007 PMC10236713

[B63] Page M. J. McKenzie J. E. Bossuyt P. M. B. I. Hoffmann T. C. Mulrow C. D. Shamseer L. . (2021). The PRISMA 2020 statement: an updated guideline for reporting systematic reviews. PLoS Med. 18:n71. 10.1136/bmj.n71 33780438 PMC8007028

[B64] Pillai J. A. Bena J. Rothenberg K. Boron B. Leverenz J. B. (2022). Association of variation in behavioral symptoms with initial cognitive phenotype in adults with dementia confirmed by neuropathology. JAMA Netw. Open 5:e220729. 10.1001/jamanetworkopen.2022.0729 35238936 PMC8895258

[B65] Rahimi J. Kovacs G. G. (2014). Prevalence of mixed pathologies in the aging brain. Alzheimers. Res. Ther. 6:82. 10.1186/s13195-014-0082-1 25419243 PMC4239398

[B66] Rascovsky K. Hodges J. R. Knopman D. Mendez M. F. Kramer J. H. Neuhaus J. . (2011). Sensitivity of revised diagnostic criteria for the behavioural variant of frontotemporal dementia. Brain 134, 2456–2477. 10.1093/brain/awr179 21810890 PMC3170532

[B67] Ropacki S. A. Jeste D. V. (2005). Epidemiology of and risk factors for psychosis of Alzheimer's disease: a review of 55 studies Published From 1990 to 2003. Am. J. Psychiat. 162, 2022–2030. 10.1176/appi.ajp.162.11.2022 16263838

[B68] Scarioni M. Gami-Patel P. Timar Y. Seelaar H. van Swieten J. C. Rozemuller A. J. M. . (2020). Frontotemporal dementia: correlations between psychiatric symptoms and pathology. Ann. Neurol. 87, 950–961. 10.1002/ana.25739 32281118 PMC7318614

[B69] Scarmeas N. Brandt J. Blacker D. Albert M. Hadjigeorgiou G. Dubois B. . (2007). Disruptive behavior as a predictor in Alzheimer disease. Arch. Neurol. 64:1755. 10.1001/archneur.64.12.1755 18071039 PMC2690610

[B70] Schaffer Aguzzoli C. Ferreira P. C. L. Povala G. Ferrari-Souza J. P. Bellaver B. Soares Katz C. . (2023). Neuropsychiatric symptoms and microglial activation in patients with Alzheimer disease. JAMA Netw. Open 6:e2345175. 10.1001/jamanetworkopen.2023.45175 38010651 PMC10682836

[B71] Sennik S. Schweizer T. A. Fischer C. E. Munoz D. G. (2016). Risk factors and pathological substrates associated with agitation/aggression in Alzheimer's disease: a preliminary study using NACC data. J. Alzheimer's Dis. 55, 1519–1528. 10.3233/JAD-160780 27911311 PMC5607738

[B72] Shaw J. S. Leoutsakos J. M. Rosenberg P. B. (2024). The relationship between first presenting neuropsychiatric symptoms in older adults and autopsy-confirmed memory disorders. Am. J. Geriatr. Psychiatry. 32, 754–764. 10.1016/j.jagp.2024.01.015 38296755 PMC11096035

[B73] Shibuya-Tayoshi S. Tsuchiya K. Seki Y. Arai T. Kasahara T. (2005). Presenile dementia mimicking Pick's disease: An autopsy case of localized amygdala degeneration with character change and emotional disorder. Neuropathology 25, 235–240. 10.1111/j.1440-1789.2005.00607.x 16193841

[B74] Staekenborg S. S. Su T. van Straaten E. C. W. Lane R. Scheltens P. Barkhof F. . (2010). Behavioural and psychological symptoms in vascular dementia; differences between small- and large-vessel disease. J. Neurol. Neurosurg. Psychiatr. 81, 547–551. 10.1136/jnnp.2009.187500 19965852

[B75] Stouffer K. M. Grande X. Düzel E. Johansson M. Creese B. Witter M. P. . (2024). Amidst an amygdala renaissance in Alzheimer's disease. Brain 147, 816–829. 10.1093/brain/awad411 38109776 PMC10907090

[B76] Sultzer D. L. Levin H. S. Mahler M. E. High W. M. Cummings J. L. (1993). A comparison of psychiatric symptoms in vascular dementia and Alzheimer's disease. Am. J. Psychiatry. 150, 1806–1812. 10.1176/ajp.150.12.1806 8238634

[B77] Sweet R. A. Hamilton R. L. Lopez O. L. Klunk W. E. Wisniewski S. R. Kaufer D. I. . (2000). Psychotic symptoms in Alzheimer's disease are not associated with more severe neuropathologic features. Int. Psychogeriatr. 12, 547–558. 10.1017/S1041610200006657 11263720

[B78] Tartaglia M. C. Kertesz A. Ang L. C. (2008). Delusions and hallucinations in frontotemporal dementia. Cogn. Behav. Neurol. 21, 107–110. 10.1097/WNN.0b013e3181799e19 18541988

[B79] Tekin S. Cummings J. L. (2002). Frontal-subcortical neuronal circuits and clinical neuropsychiatry: an update. J. Psychosom. Res. 53, 647–654. 10.1016/S0022-3999(02)00428-2 12169339

[B80] Tekin S. Mega M. S. Masterman D. M. Chow T. Garakian J. Vinters H. V. . (2001). Orbitofrontal and anterior cingulate cortex neurofibrillary tangle burden is associated with agitation in Alzheimer disease. Ann. Neurol. 49, 355–361. 10.1002/ana.72 11261510

[B81] Tissot C. Therriault J. Pascoal T. A. Chamoun M. Lussier F. Z. Savard M. . (2021). Association between regional tau pathology and neuropsychiatric symptoms in aging and dementia due to Alzheimer's disease. Alzheimer's Dement. 7:e12154. 10.1002/trc2.12154 33816761 PMC8012244

[B82] Tsuang D. Larson E. B. Bolen E. Thompson M. Lou Peskind E. Bowen J. . (2009). Visual hallucinations in dementia: a prospective community-based study with autopsy. Am. J. Geriatr. Psychiat. 17, 317–323. 10.1097/JGP.0b013e3181953b9a 19307860 PMC2742470

[B83] Van der Linde R. M. Dening T. Matthews F. E. Brayne C. (2014). Grouping of behavioural and psychological symptoms of dementia. Int. J. Geriatr. Psychiat. 29, 562–568. 10.1002/gps.4037 24677112 PMC4255309

[B84] Van der Linde R. M. Dening T. Stephan B. C. M. Prina A. M. Evans E. Brayne C. . (2016). Longitudinal course of behavioural and psychological symptoms of dementia: systematic review. Br. J. Psychiat. 209, 366–377. 10.1192/bjp.bp.114.148403 27491532 PMC5100633

[B85] Victoroff J. Zarow C. Mack W. J. Hsu E. Chui H. C. (1996). Physical aggression is associated with preservation of substantia nigra pars compacta in Alzheimer disease. Arch. Neurol. 53, 428–434. 10.1001/archneur.1996.00550050058024 8624218

[B86] Vik-Mo A. O. Bencze J. Ballard C. Hortobágyi T. Aarsland D. (2019). Advanced cerebral amyloid angiopathy and small vessel disease are associated with psychosis in Alzheimer's disease. J. Neurol. Neurosurg. Psychiatr. 90, 728–730. 10.1136/jnnp-2018-318445 30054314

[B87] Vogel J. W. Young A. L. Oxtoby N. P. Smith R. Ossenkoppele R. Strandberg O. T. . (2021). Four distinct trajectories of tau deposition identified in Alzheimer's disease. Nat. Med. 27, 871–881. 10.1038/s41591-021-01309-6 33927414 PMC8686688

[B88] World Health Organization (2024). The epidemiology and impact of dementia: current state and future trends. Available at: https://www.who.int/news-room/fact-sheets/detail/dementiadementia (accessed August 10, 2024).

[B89] Zhao K. Xie H. Fonzo G. A. Carlisle N. B. Osorio R. S. Zhang Y. . (2024). Dementia subtypes defined through neuropsychiatric symptom–associated brain connectivity patterns. JAMA Netw. Open 7:e2420479. 10.1001/jamanetworkopen.2024.20479 38976268 PMC11231801

[B90] Zhao Q. F. Tan L. Wang H. F. Jiang T. Tan M. S. Tan L. . (2016a). The prevalence of neuropsychiatric symptoms in Alzheimer's disease: Systematic review and meta-analysis. J. Affect. Disord. 190, 264–271. 10.1016/j.jad.2015.09.069 26540080

[B91] Zhao Q. F. Tan L. Wang H. F. Jiang T. Tan M. S. Tan L. . (2016b). Corrigendum to: “The prevalence of neuropsychiatric symptoms in Alzheimer's disease: Systematic review and meta-analysis” [J. Affect. Disord. 190 (2016) 264–271]. J. Affect. Disord. 206:8. 10.1016/j.jad.2016.04.054 27455352

[B92] Zubenko G. S. Moossy J. Martinez A. J. Rao G. Claassen D. Rosen J. . (1991). Neuropathologic and neurochemical correlates of psychosis in primary dementia. Arch. Neurol. 48, 619–624. 10.1001/archneur.1991.00530180075020 1710105

