# Supplementary material for: Investigating neuropathological correlates of hyperactive and psychotic symptoms in dementia: a systematic review
Source: Front Dement. 2025 Jan 29;4:1513644. doi: 10.3389/frdem.2025.1513644 (PMC11814221; doi:10.3389/frdem.2025.1513644)
Supplement: Supplementary file 1 [file Table_1.docx]

Supplementary Material

**Appendix I.** Search strategy.

PubMed search strategy:

((behavioral symptom*) OR (psychological symptom*) OR (behavioral and psychological symptom*) OR (BPSD) OR (neuropsychiatric symptom*) OR (behavioral symptoms [MeSH Terms])) AND ((agitation) OR (hyperactiv*) OR (delusions [MeSH Terms]) OR (delusion*) OR (aberrant motor behavior in dementia [MeSH Terms]) OR (aggression [MeSH Terms]) OR (aggression) OR (hallucinations [MeSH Terms]) OR (HIDA) OR (anxiety) OR (disinhibit*) OR (irritability) OR (irritative behavior [MeSH Terms]) OR (impulsivity) OR (impulsive behavior [MeSH Terms]) or (wandering)) AND ((neuropatholog*) OR (synucleinopath*) OR (tauopath*) OR (TDP-43 proteinopath*) OR (proteinopath*) OR (amyloidopath*)) AND ((neurodegenerative disease [Mesh Terms]) OR (neurocognitive disorder*) OR (cognitive disorder*) OR (dementia*) OR (cognitive decline) OR (neurodegeneration) OR (dementia [MeSH Terms]))

EMBASE search strategy:

('behavioral symptom*' OR 'psychological symptom*' OR 'behavioral and psychological symptom*' OR 'bpsd' OR 'neuropsychiatric symptom*') AND ('agitation' OR 'hyperactiv*' OR 'delusion*' OR 'aberrant motor behavior' OR 'aggression' OR 'aggressive*' OR 'hallucination*' OR 'hida' OR 'anxiety' OR 'disinhibit*' OR 'irritability' OR 'irritative*' OR 'impulsiveness' OR 'impulsivity' OR 'impulsive*' OR 'wandering') AND (‘neuropatholog*’ OR ‘synucleinopath*’ OR ‘tauopath*’ OR ‘TDP-43 proteinopath*’ OR ‘proteinopath*’ OR ‘amyloidopath*’) AND ('neurocognitive disorder*' OR 'cognitive disorder*' OR 'dementia' OR 'cognitive declin*' OR 'neurodegeneration' OR 'neurodegenerative disease*' OR 'alzheimer*' OR 'lewy bod*' OR 'vascular dement*' OR 'mixed dement*')

Cochrane Library search strategy:

((behavioral symptom*) OR (psychological symptom*) OR (behavioral and psychological symptom*) OR (BPSD) OR (neuropsychiatric symptom*)) AND ((agitation) OR (hyperactiv*) OR (delusion*) OR (aberrant motor behavior) OR (aggression) OR (aggressive*) OR (hallucination*) OR (HIDA) OR (anxiety) OR (disinhibit*) OR (irritability) OR (irritative) OR (irritable) OR (impulsivity) OR (impulsive*) or (wandering)) AND ((neuropatholog*) OR (synucleinopath*) OR (tauopath*) OR (TDP-43 proteinopath*) OR (proteinopath*) OR (amyloidopath*)) AND ((cognitive disorder*) OR (dement*) OR (cognitive declin*) OR (neurodegeneration) OR (neurodegenerative disease*) OR (alzheimer disease*) OR (frontotemporal dement*) OR (lewy body disease))

**Table S1.** PRISMA Checklist 2020. From Page MJ, McKenzie JE, Bossuyt PM, Boutron I, Hoffmann TC, Mulrow CD, et al. The PRISMA 2020 statement: an updated guideline for reporting systematic reviews. BMJ 2021;372:n71. doi: 10.1136/bmj.n71.

| **Section and Topic** | **Item #** | **Checklist item** | **Location where item is reported** |
| --- | --- | --- | --- |
| **TITLE** | | |  |
| Title | 1 | Identify the report as a systematic review. | 1 |
| **ABSTRACT** | | |  |
| Abstract | 2 | See the PRISMA 2020 for Abstracts checklist. | 1, 2 |
| **INTRODUCTION** | | |  |
| Rationale | 3 | Describe the rationale for the review in the context of existing knowledge. | 2, 3 |
| Objectives | 4 | Provide an explicit statement of the objective(s) or question(s) the review addresses. | 3 |
| **METHODS** | | |  |
| Eligibility criteria | 5 | Specify the inclusion and exclusion criteria for the review and how studies were grouped for the syntheses. | 4 |
| Information sources | 6 | Specify all databases, registers, websites, organisations, reference lists and other sources searched or consulted to identify studies. Specify the date when each source was last searched or consulted. | 3, 4 |
| Search strategy | 7 | Present the full search strategies for all databases, registers and websites, including any filters and limits used. | 3, 4 |
| Selection process | 8 | Specify the methods used to decide whether a study met the inclusion criteria of the review, including how many reviewers screened each record and each report retrieved, whether they worked independently, and if applicable, details of automation tools used in the process. | 4 |
| Data collection process | 9 | Specify the methods used to collect data from reports, including how many reviewers collected data from each report, whether they worked independently, any processes for obtaining or confirming data from study investigators, and if applicable, details of automation tools used in the process. | 4 |
| Data items | 10a | List and define all outcomes for which data were sought. Specify whether all results that were compatible with each outcome domain in each study were sought (e.g. for all measures, time points, analyses), and if not, the methods used to decide which results to collect. | 4 |
|  | 10b | List and define all other variables for which data were sought (e.g. participant and intervention characteristics, funding sources). Describe any assumptions made about any missing or unclear information. | 4 |
| Study risk of bias assessment | 11 | Specify the methods used to assess risk of bias in the included studies, including details of the tool(s) used, how many reviewers assessed each study and whether they worked independently, and if applicable, details of automation tools used in the process. | 4 |
| Effect measures | 12 | Specify for each outcome the effect measure(s) (e.g. risk ratio, mean difference) used in the synthesis or presentation of results. | 4 |
| Synthesis methods | 13a | Describe the processes used to decide which studies were eligible for each synthesis (e.g. tabulating the study intervention characteristics and comparing against the planned groups for each synthesis (item #5)). | N/A |
|  | 13b | Describe any methods required to prepare the data for presentation or synthesis, such as handling of missing summary statistics, or data conversions. | N/A |
|  | 13c | Describe any methods used to tabulate or visually display results of individual studies and syntheses. | N/A |
|  | 13d | Describe any methods used to synthesize results and provide a rationale for the choice(s). If meta-analysis was performed, describe the model(s), method(s) to identify the presence and extent of statistical heterogeneity, and software package(s) used. | 4 |
|  | 13e | Describe any methods used to explore possible causes of heterogeneity among study results (e.g. subgroup analysis, meta-regression). | N/A |
|  | 13f | Describe any sensitivity analyses conducted to assess robustness of the synthesized results. | N/A |
| Reporting bias assessment | 14 | Describe any methods used to assess risk of bias due to missing results in a synthesis (arising from reporting biases). | 4 |
| Certainty assessment | 15 | Describe any methods used to assess certainty (or confidence) in the body of evidence for an outcome. | N/A |
| **RESULTS** | | |  |
| Study selection | 16a | Describe the results of the search and selection process, from the number of records identified in the search to the number of studies included in the review, ideally using a flow diagram. | 4 |
|  | 16b | Cite studies that might appear to meet the inclusion criteria, but which were excluded, and explain why they were excluded. | 4 |
| Study characteristics | 17 | Cite each included study and present its characteristics. | 5 |
| Risk of bias in studies | 18 | Present assessments of risk of bias for each included study. | Supplementary Materials |
| Results of individual studies | 19 | For all outcomes, present, for each study: (a) summary statistics for each group (where appropriate) and (b) an effect estimate and its precision (e.g. confidence/credible interval), ideally using structured tables or plots. | 24-27 |
| Results of syntheses | 20a | For each synthesis, briefly summarise the characteristics and risk of bias among contributing studies. | 4, 5 |
|  | 20b | Present results of all statistical syntheses conducted. If meta-analysis was done, present for each the summary estimate and its precision (e.g. confidence/credible interval) and measures of statistical heterogeneity. If comparing groups, describe the direction of the effect. | 5, 6, 7 |
|  | 20c | Present results of all investigations of possible causes of heterogeneity among study results. | N/A |
|  | 20d | Present results of all sensitivity analyses conducted to assess the robustness of the synthesized results. | N/A |
| Reporting biases | 21 | Present assessments of risk of bias due to missing results (arising from reporting biases) for each synthesis assessed. | N/A |
| Certainty of evidence | 22 | Present assessments of certainty (or confidence) in the body of evidence for each outcome assessed. | N/A |
| **DISCUSSION** | | |  |
| Discussion | 23a | Provide a general interpretation of the results in the context of other evidence. | 7-13 |
|  | 23b | Discuss any limitations of the evidence included in the review. | 7, 8 |
|  | 23c | Discuss any limitations of the review processes used. | 7 |
|  | 23d | Discuss implications of the results for practice, policy, and future research. | N/A |
| **OTHER INFORMATION** | | |  |
| Registration and protocol | 24a | Provide registration information for the review, including register name and registration number, or state that the review was not registered. | 14 |
|  | 24b | Indicate where the review protocol can be accessed, or state that a protocol was not prepared. | 14 |
|  | 24c | Describe and explain any amendments to information provided at registration or in the protocol. | N/A |
| Support | 25 | Describe sources of financial or non-financial support for the review, and the role of the funders or sponsors in the review. | 14 |
| Competing interests | 26 | Declare any competing interests of review authors. | 14 |
| Availability of data, code and other materials | 27 | Report which of the following are publicly available and where they can be found: template data collection forms; data extracted from included studies; data used for all analyses; analytic code; any other materials used in the review. | 14 |

**Appendix II.** Quality assessment of the included studies.

**Table S2a.** Visual representation of the quality assessment of included case-control studies using the Newcastle-Ottawa Scale (NOS).

| **Study** | **Selection** | | | | **Comparability** | | **Exposure** | | | **Final score** |
| --- | --- | --- | --- | --- | --- | --- | --- | --- | --- | --- |
|  | **Adequate case definition** | **Representativeness of cases** | **Selection**  **of controls** | **Definition of controls** | **Main factor** | **Additional factors** | **Ascertainment of exposure** | **Same method of ascertainment for cases and controls** | **Non-response rate** |  |
| Ferman et al. (57) | ✓ | ✓ |  | ✓ | ✓ | ✓ | ✓ | ✓ | ✓ | 8/9 |
| Forstl et al. (59) | ✓ | ✓ | ✓ | ✓ | ✓ | ✓ | ✓ | ✓ | ✓ | 9/9 |
| Harding et al. (53) | ✓ | ✓ | ✓ | ✓ | ✓ | ✓ | ✓ | ✓ |  | 8/9 |
| Leger et al. (58) | ✓ | ✓ | ✓ | ✓ | ✓ |  | ✓ | ✓ | ✓ | 8/9 |
| Murray et al. (31) | ✓ | ✓ | ✓ | ✓ | ✓ | ✓ | ✓ | ✓ | ✓ | 9/9 |
| Nelson et al. (49) | ✓ | ✓ | ✓ | ✓ | ✓ | ✓ | ✓ | ✓ | ✓ | 9/9 |
| Sennik et al. (28) | ✓ | ✓ | ✓ | ✓ | ✓ |  | ✓ | ✓ | ✓ | 8/9 |
| Sweet et al. (46) | ✓ | ✓ | ✓ | ✓ | ✓ | ✓ | ✓ | ✓ | ✓ | 9/9 |
| Victoroff et al. (29) | ✓ | ✓ | ✓ | ✓ | ✓ | ✓ | ✓ | ✓ | ✓ | 9/9 |
| Zubenko et al. (61) | ✓ | ✓ | ✓ | ✓ | ✓ | ✓ | ✓ | ✓ | ✓ | 9/9 |

✓ = condition satisfied

**Table S2b.** Visual representation of the quality assessment of included cohort studies using the Newcastle-Ottawa Scale (NOS).

| **Study** | **Selection** | | | | **Comparability** | | **Exposure** | | | **Final score** |
| --- | --- | --- | --- | --- | --- | --- | --- | --- | --- | --- |
|  | **Representativeness of the exposed cohort** | **Selection of the non-exposed cohort** | **Ascertainment of exposure** | **Outcome of interest absent at study initiation** | **Main factor** | **Additional factors** | **Assessment of outcome** | **Adequacy of follow-up duration** | **Adequacy of follow up of cohorts** |  |
| Ballard et al. (88) | ✓ | ✓ | ✓ | ✓ | ✓ | ✓ | ✓ | ✓ | ✓ | 9/9 |
| Borges et al. (54) | ✓ | ✓ | ✓ | ✓ | ✓ | ✓ | ✓ | ✓ | ✓ | 9/9 |
| Denevand et al. (51) | ✓ | ✓ | ✓ | ✓ | ✓ | ✓ | ✓ | ✓ | ✓ | 9/9 |
| Esteban de Antonio et al. (40) | ✓ |  | ✓ | ✓ | ✓ | ✓ | ✓ | ✓ |  | 7/9 |
| Hirsch-Reinshagen et al. (90) | ✓ | ✓ | ✓ | ✓ | ✓ | ✓ | ✓ | ✓ | ✓ | 9/9 |
| Lai et al. (60) | ✓ |  | ✓ | ✓ | ✓ | ✓ | ✓ | ✓ |  | 7/9 |
| Pillai et al. (44) | ✓ | ✓ | ✓ | ✓ | ✓ | ✓ | ✓ | ✓ | ✓ | 9/9 |
| Scarioni et al. (70) | ✓ |  | ✓ | ✓ | ✓ | ✓ | ✓ | ✓ | ✓ | 8/9 |
| Shaw et al. (52) | ✓ | ✓ | ✓ | ✓ | ✓ | ✓ | ✓ | ✓ |  | 8/9 |
| Vik-Mo et al. (79) | ✓ | ✓ | ✓ | ✓ | ✓ | ✓ | ✓ | ✓ | ✓ | 9/9 |

✓ = condition satisfied

**Table S2c.** Visual representation of the quality assessment of included cross-sectional studies using the adapted Newcastle-Ottawa Scale (NOS).

| **Study** | **Selection** | | | | **Comparability** | | **Outcome** | | **Final score** |
| --- | --- | --- | --- | --- | --- | --- | --- | --- | --- |
|  | **Sample representativeness** | **Sample size** | **Non-respondents** | **Validity of exposure or outcome** | **Main factor** | **Additional factors** | **Outcome assessment** | **Statistical analysis** |  |
| Echavarri et al. (30) | ✓ |  | ✓ | ✓ | ✓ | ✓ | ✓ |  | 6/8 |
| Ehrenberg et al. (63) | ✓ |  | ✓ | ✓ | ✓ | ✓ | ✓ | ✓ | 7/8 |
| Farber et al. (45) | ✓ |  | ✓ | ✓ | ✓ | ✓ | ✓ | ✓ | 7/8 |
| Garcia-Segura et al. (89) | ✓ |  | ✓ | ✓ | ✓ | ✓ | ✓ | ✓ | 7/8 |
| Gauthreaux et al. (41) | ✓ |  | ✓ | ✓ | ✓ | ✓ | ✓ |  | 6/8 |
| Gibson et al. (50) | ✓ |  | ✓ | ✓ | ✓ | ✓ | ✓ | ✓ | 7/8 |
| Liu et al. (48) | ✓ |  | ✓ | ✓ | ✓ |  | ✓ |  | 5/8 |
| Tekin et al. (62) | ✓ |  | ✓ | ✓ |  |  | ✓ |  | 4/8 |
| Tsuang et al. (72) | ✓ |  | ✓ | ✓ | ✓ |  | ✓ | ✓ | 6/8 |

✓ = condition satisfied

**Table S2d.** Visual representation of the quality assessment of included case reports and case series using the CARE guidelines.

| **Study** | **Title** | **Key words** | **Abstract** | **Introduction** | **Patient information** | **Clinical findings** | **Timeline** | **Diagnostic Assessment** | **Therapeutic intervention** | **Follow-up and outcomes** | **Discussion** | **Patient perspective** | **Inform consent** | **Final score** |
| --- | --- | --- | --- | --- | --- | --- | --- | --- | --- | --- | --- | --- | --- | --- |
| Asaoka et al. (34) |  | ✓ | ✓ ✓ | ✓ | ✓ | ✓ | ✓ | ✓ |  | ✓ ✓ | ✓ ✓ |  |  | 12/19 |
| Beck et al. (81) | ✓ | ✓ | ✓ | ✓ | ✓ ✓ | ✓ | ✓ | ✓ ✓ | ✓ | ✓ | ✓ |  |  | 13/19 |
| Fujishiro et al. (32) |  | ✓ | ✓ ✓ | ✓ | ✓ ✓ | ✓ | ✓ | ✓ ✓ | ✓ | ✓ | ✓ ✓ |  |  | 14/19 |
| Hirano et al. (91) |  | ✓ | ✓ ✓ | ✓ | ✓ | ✓ | ✓ | ✓ ✓ | ✓ | ✓ | ✓ ✓ |  |  | 13/19 |
| Iwasaki et al. (82) |  | ✓ | ✓ | ✓ | ✓ ✓ | ✓ | ✓ | ✓ ✓ | ✓ | ✓ | ✓ ✓ |  |  | 13/19 |
| Kawakami et al. (92) | ✓ | ✓ | ✓ ✓ | ✓ | ✓ ✓ | ✓ | ✓ | ✓ ✓ | ✓ ✓ | ✓ ✓ | ✓ ✓ |  | ✓ | 18/19 |
| Shibuya-Tayoshi et al. (33) | ✓ | ✓ | ✓ ✓ | ✓ | ✓ ✓ | ✓ | ✓ | ✓ |  | ✓ ✓ | ✓ ✓ |  |  | 14/19 |
| Tartaglia et al. (93) | ✓ | ✓ | ✓ | ✓ | ✓ | ✓ | ✓ | ✓ |  | ✓ | ✓ ✓ |  |  | 11/19 |

✓ = condition partially satisfied

✓ ✓ = condition satisfied
